# Supplementary material for: Spatial analysis of the glioblastoma proteome reveals specific molecular signatures and markers of survival
Source: Nat Commun. 2022 Nov 4;13:6665. doi: 10.1038/s41467-022-34208-6 (PMC9636229; doi:10.1038/s41467-022-34208-6)
Supplement: Supplementary file 1 — Supplementary Information [file 41467_2022_34208_MOESM1_ESM.pdf]

Supplementary Information

Spatial analysis of the glioblastoma proteome reveals specific molecular signatures and markers of survival

Marie Duhamel[1§], Lauranne Drelich[1§], Maxence Wisztorski[1§], Soulaimane Aboulouard[1], Jean-Pascal Gimeno[1], Nina Ogrinc[1], Patrick Devos[2], Tristan Cardon[1], Michael Weller[3], Fabienne Escande[4], Fahed Zairi[5], Claude-Alain Maurage[4], Émilie Le Rhun[1,3,4*], Isabelle Fournier[1,6*]and Michel Salzet[1,6*]

[1]Univ.Lille, Inserm, CHU Lille, U1192, Laboratoire Protéomique, Réponse Inflammatoire et Spectrométrie de Masse (PRISM), F-59000 Lille, France

[2] Univ. Lille, CHU Lille, ULR 2694 - METRICS : Évaluation des technologies de santé et des pratiques médicales, F-59000 Lille, France

[3] Department of Neurology & Clinical Neuroscience Center, University Hospital and University of Zurich, Zurich, Switzerland

[4] CHU Lille, Service de biochimie et biologie moléculaire, CHU Lille, F-59000 Lille France

[5] CHU Lille, Service de neurochirurgie, F-59000 Lille, France

[6]Institut Universitaire de France (IUF), Paris, 75000, France

[§] These authors contributed equally Marie Duhamel, Lauranne Drelich, Maxence Wisztorski

* These authors jointly supervised this work Emilie Le Rhun, Isabelle Fournier, Michel Salzet

Corresponding authors

Correspondence to Marie Duhamel (marie.duhamel@univ-lille.fr), Emilie Le Rhun (emilie.lerhun@usz.ch), , Isabelle Fournier (isabelle.fournier@univ-lille.fr) and Michel Salzet (michel.salzet@univ-lille.fr)

Supplementary Table 1. Model cross-validation from the SpiderMass recorded in the positive ion mode from 30 different tumors using the "20% randomly taken" and the "1 patient out" methods. Percentage of good classifications are given including or excluding outliers.

Model type	ion mode	n classes	n tested spectra	n passes	n failures	N outliers	Correct classification rate (%)	
							Excluding outliers	Including outliers
**Cross Validation by 20% randomly taken**								
Glioma	Positive	3	135	124	0	11	100%	91.85%
**Cross Validation by 1 patient out method**								
Glioma	Positive	3	135	105	8	22	92.92%	77.78%

Supplementary Table 2. Histological composition of each of the three regions identified by MALDI-MSI. The percentage of micro-extracted points in the histological areas was calculated for each molecular region (yellow-B, red-A, and blue-C).

Region	**Tumor**	**Very dense infiltration**	**dense infiltration**	**necrosis+ tumor**	**MVP+ tumor**	**tumor+ inflammation**	**necrosis**
**yellow**	45%	0%	0%	25%	5%	0%	20%
**red**	39.3%	14.3%	28.6%	10.7%	3.6%	3.6%	0%
**blue**	73.6%	5.7%	2.3%	3.4%	12.6%	1.1%	1.1%

Supplementary Table 3. List of the proteomics extraction points and their associated region

Extraction point	Region	Extraction point	Region	Extraction point	Region
12-1	A	48-1	B	34-2	C
12-2	A	53-1	B	34-3	C
12-3	A	9-2	B	35-1	C
12-4	A	10-1	C	35-2	C
14-1	A	10-2	C	35-3	C
14-2	A	1-1	C	36-3	C
14-3	A	1-2	C	37-1	C
20-1	A	13-1	C	38-2	C
20-2	A	13-2	C	39-1	C
20-3	A	13-3	C	39-3	C
20-4	A	15-1	C	39-4	C
21-1	A	15-2	C	4-1	C
21-2	A	15-3	C	41-1	C
25-1	A	16-1	C	41-2	C
25-2	A	16-2	C	41-3	C
25-3	A	16-3	C	42-2	C
31-1	A	17-1	C	42-3	C
31-2	A	17-2	C	43-1	C
31-3	A	17-3	C	44-1	C
32-1	A	17-4	C	44-2	C
36-1	A	19-1	C	44-3	C
36-2	A	19-2	C	45-1	C
43-2	A	2-1	C	45-2	C
46-3	A	22-1	C	46-1	C
49-3	A	22-2	C	46-2	C
49-4	A	2-3	C	47-1	C
5-1	A	24-1	C	47-2	C
5-2	A	24-2	C	48-2	C
1-3	B	24-3	C	48-3	C
2-2	B	24-4	C	49-1	C
26-1	B	25-4	C	49-2	C
27-1	B	25-5	C	50-1	C
27-2	B	26-2	C	50-2	C
27-3	B	28-1	C	50-3	C
32-2	B	28-2	C	51-1	C
32-3	B	29-1	C	51-2	C
37-2	B	29-2	C	51-3	C
37-3	B	29-3	C	53-2	C

38-1	B	30-1	C	53-3	C
38-3	B	30-2	C	6-1	C
39-2	B	33-1	C	8-1	C
4-2	B	33-2	C	8-2	C
42-1	B	33-3	C	8-3	C
47-3	B	33-4	C	9-1	C
47-4	B	34-1	C	9-3	C

Supplementary Table 4. Ghost proteins overexpressed in the 3 regions A B C

GROUP	protein accession	protein length (a.a.)	molecular weight (kDa)	isoelectric point	gene symbol	type	localization
C	IP_063564	113	12.39	9.26	*CLDN19*	mRNA	3'UTR
C	IP_065285	48	5.17	8.97	*C8B*	mRNA	CDS
C	IP_072691	36	4.26	8.51	*CD84*	mRNA	3'UTR
C	IP_073718	85	9.63	10.84	*CCDC181*	mRNA	CDS
C	IP_079312	388	42.34	5.71	*EDARADD*	mRNA	3'UTR
C	IP_092740	46	5.08	10.69	*TTN*	mRNA	CDS
C	IP_219633	35	3.95	5.67	*POSTN*	mRNA	3'UTR
C	IP_2277691	46	5.53	8.78	*RTTN*	mRNA	CDS
C	IP_2303139	56	6.24	6.49	*LOC105369735*	ncRNA	-
C	IP_2323408	61	6.86	8.55	*LOC105376126*	ncRNA	-
C	IP_2348387	31	3.34	7.26	*COL19A1*	mRNA	5'UTR
C	IP_235425	38	4.24	10.31	*HCN4*	mRNA	3'UTR
C	IP_2376460	57	6.73	7.36	*EPHA6*	mRNA	3'UTR
C	IP_2393992	43	5.22	11.65	*LOC105376830*	ncRNA	-
C	IP_256988	39	4.52	9.45	*TBX21*	mRNA	3'UTR
C	IP_261897	41	4.44	9.67	*AC108004.2*	ncRNA	-
C	IP_557239	90	10.19	9.53	*TUBB4AP1*	ncRNA	-
C	IP_559054	30	3.56	4.27	*SLITRK2*	mRNA	5'UTR
C	IP_563986	424	47.8	4.43	*KRT8P11*	ncRNA	-
C	IP_572422	212	24.18	4.64	*TUBBP1*	ncRNA	-
C	IP_580018	247	29.04	11.36	*RPL7P32*	ncRNA	-
C	IP_590938	74	8.75	10.05	*HMGB3P18*	ncRNA	-
C	IP_629960	30	3.57	9.69	*LRRC34*	ncRNA	-
C	IP_669601	75	8.32	10.63	*HIST2H2BB*	ncRNA	-
C	IP_671464	249	26.94	8.21	*TPI1P1*	ncRNA	-
C	IP_701463	179	20.51	9.05	*CTD-2541J13.2*	ncRNA	-
C	IP_739889	139	15.18	5.67	*GOLGA6L9*	ncRNA	-
C	IP_752010	59	6.39	10.62	*MIPOL1*	mRNA	3'UTR
C	IP_774693	75	8.68	3.92	*TUBAP2*	ncRNA	-
C	IP_774695	374	41.01	5.68	*TUBAP2*	ncRNA	-
A	IP_2390879	36	4.08	5.49	*LOC107985743*	ncRNA	-
A	IP_244732	78	8.56	7.13	*KIFC3*	mRNA	5'UTR
B	IP_156671	120	14.22	10.43	*SLC13A1*	mRNA	3'UTR
B	IP_222588	58	6.32	11.27	*CPB2-AS1*	ncRNA	-
B	IP_2389079	29	2.98	8.35	*MYT1L*	mRNA	5'UTR
B	IP_265416	32	3.81	10.78	*LIPG*	mRNA	CDS
B	IP_273562	76	8.34	11.89	*LGI4*	mRNA	5'UTR
B	IP_278725	69	7.77	10.83	*ZNF888*	mRNA	CDS

B	IP_592742	86	9.64	5.86	*KRT18P64*	ncRNA	-
B	IP_602497	38	4.24	5.65	*CTD-2151A2.2*	ncRNA	-
B	IP_672223	178	20.37	5.14	*GBP1P1*	ncRNA	-

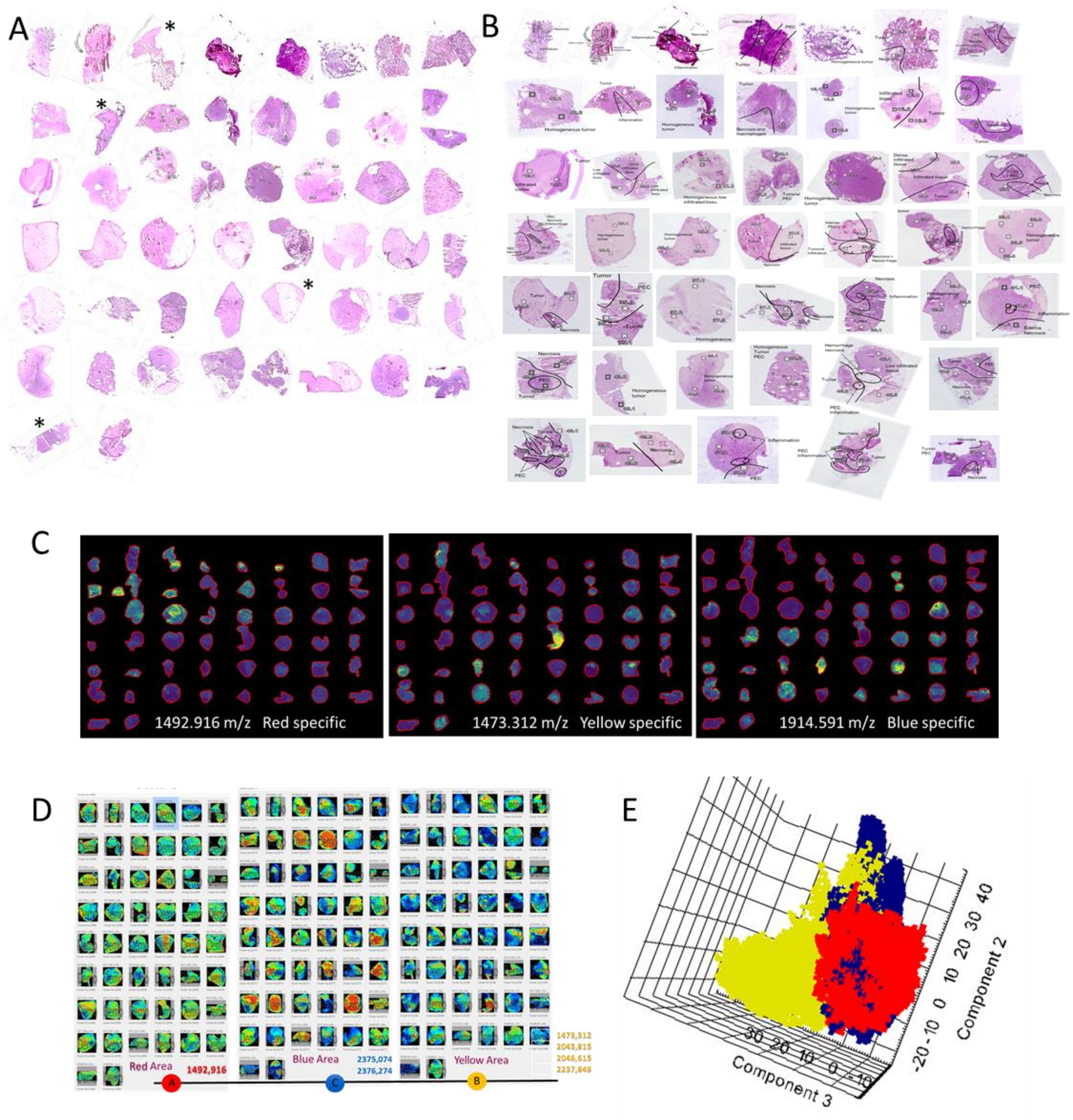

Supplementary Figure 1. Classification of the 46 tumors by MALDI-MSI and relation to the pathologist annotations.

A. Scanned pictures after hematoxylin-eosin staining of the 46 glioblastoma tumors

B. Pathologist annotations of the glioblastoma samples. The pathologist annotated each sample's different regions of interest (tumors, endothelial proliferation, necrosis, infiltration, blood...).

C. MALDI MSI images of characteristics m/z ions for each group.

D. Ward clustering method gives 3 main branches with the same characteristic ions.

E. Principal component analysis (PCA) of each spectra reveals separation between the three regions.

1

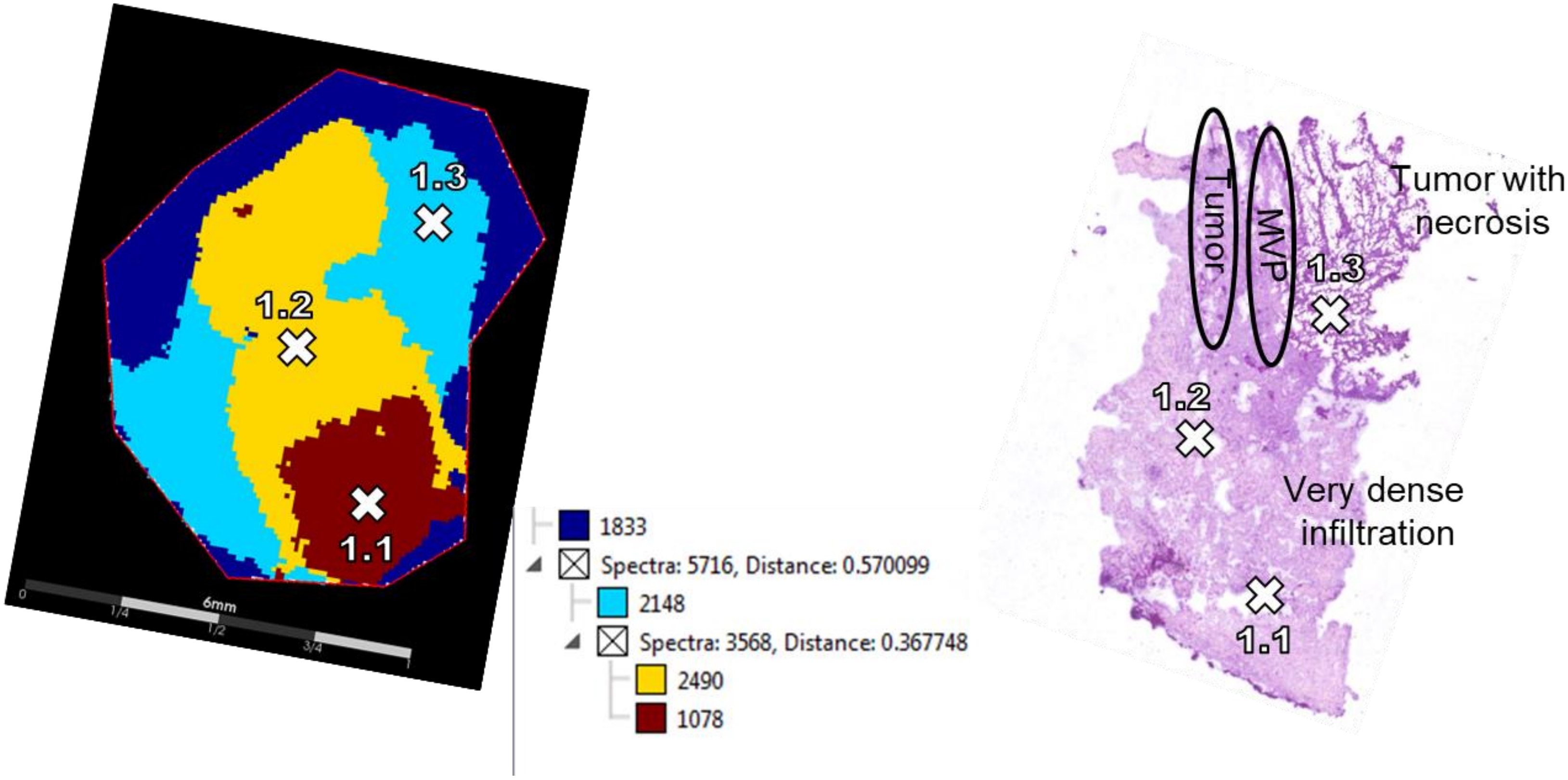
1.3
1.2
1.1
6mm
0
1/4
1/2
3/4
1
1833
Spectra: 5716, Distance: 0.570099
2148
Spectra: 3568, Distance: 0.367748
2490
1078
Tumor
MVP
Tumor with necrosis
1.3
1.2
Very dense infiltration
1.1

2

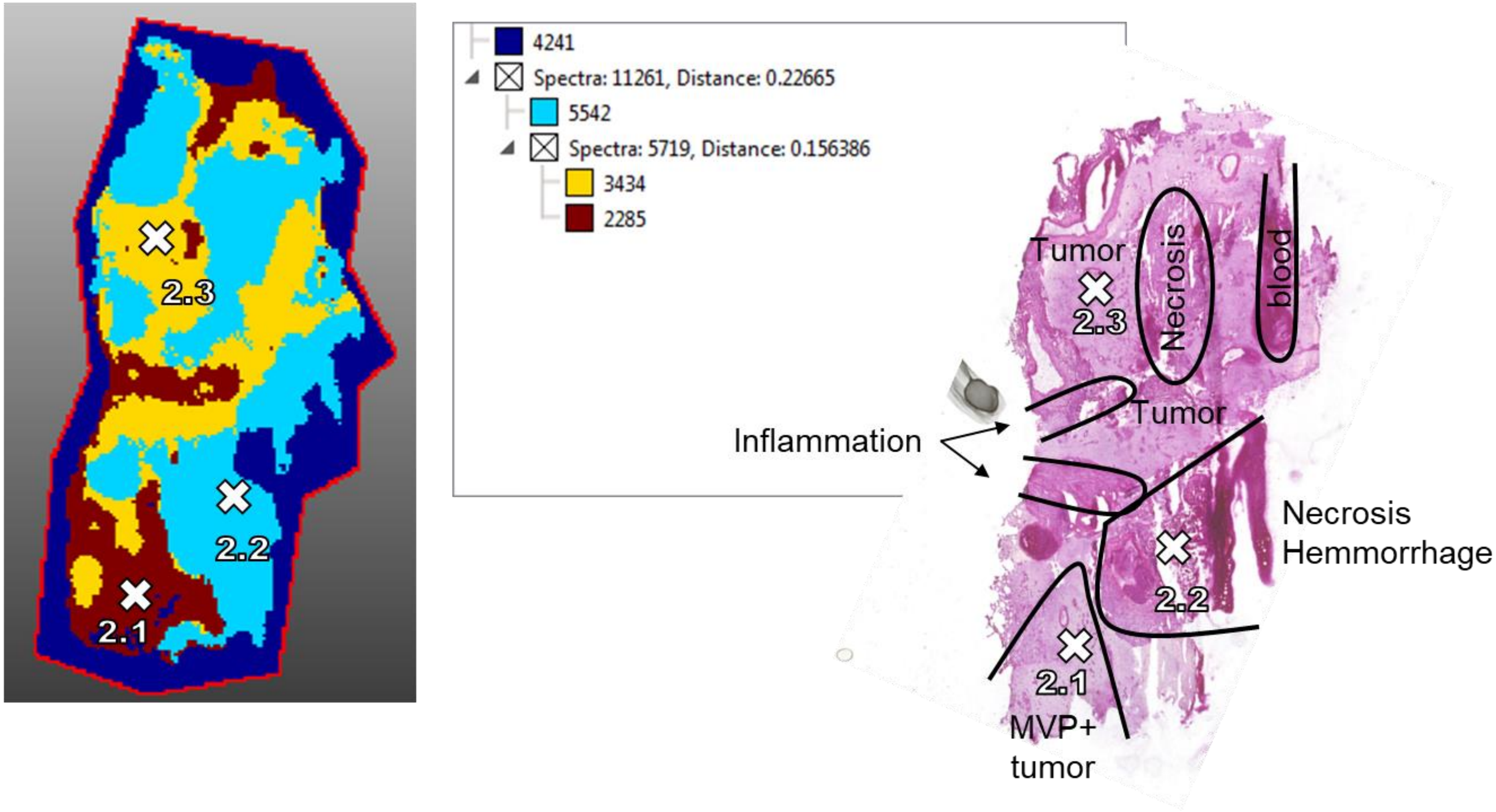
2.3
2.2
2.1
4241
Spectra: 11261, Distance: 0.22665
5542
Spectra: 5719, Distance: 0.156386
3434
2285
Tumor
2.3
Necrosis
blood
Inflammation
Tumor
Necrosis
Hemmorrhage
2.2
2.1
MVP+
tumor

3

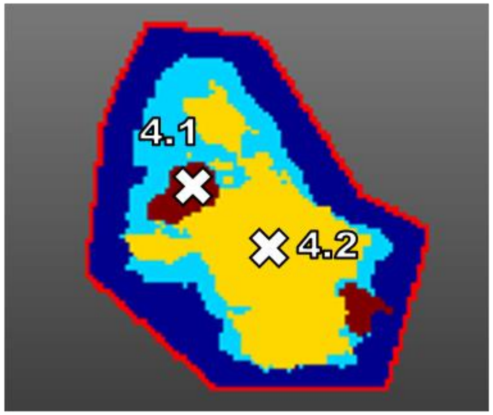
4.1
4.2

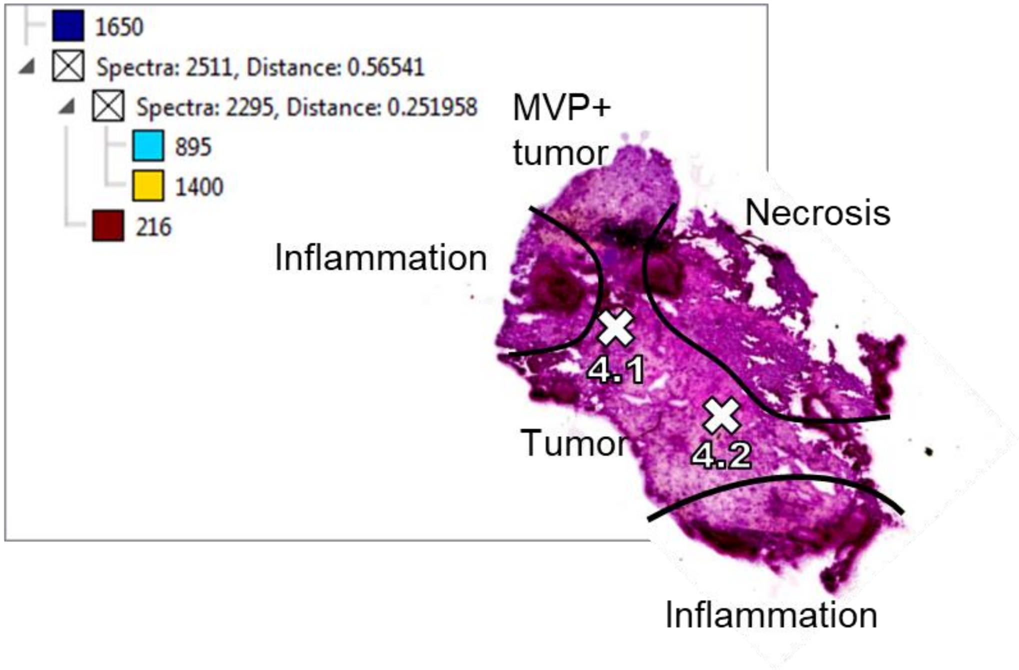
1650
Spectra: 2511, Distance: 0.56541
Spectra: 2295, Distance: 0.251958
895
1400
216
MVP+
tumor
Necrosis
Inflammation
4.1
Tumor
4.2
Inflammation

4

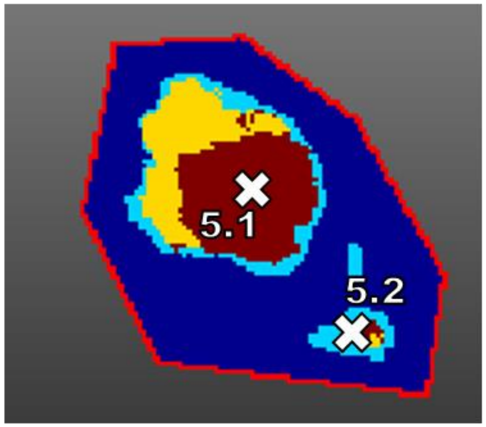

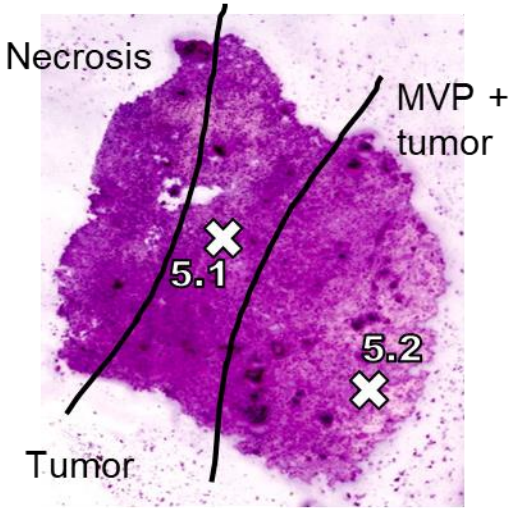

5

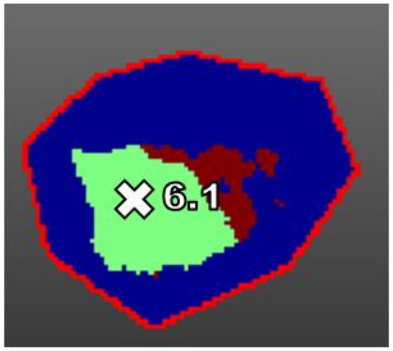

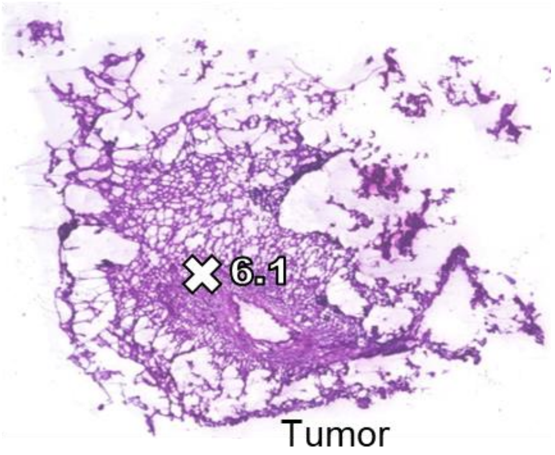

6

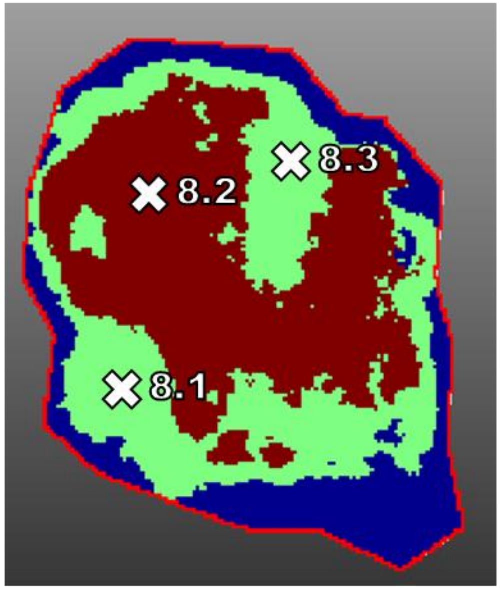

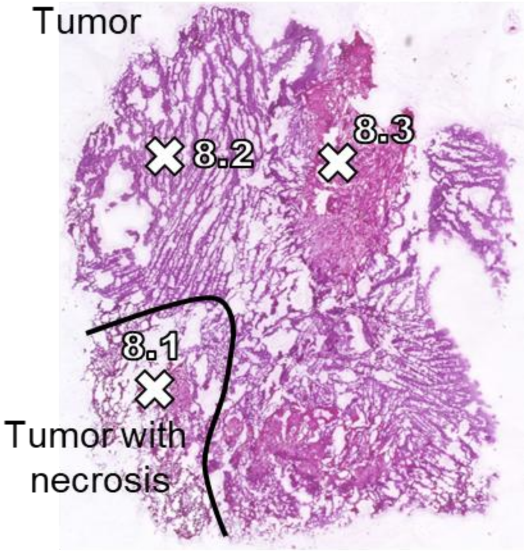

7

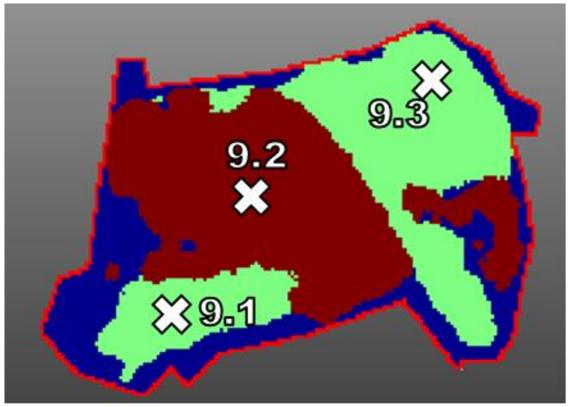

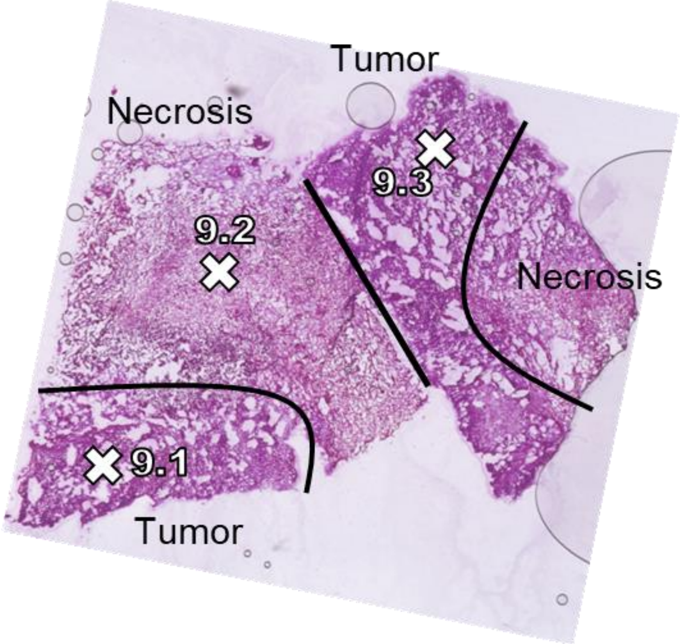

8

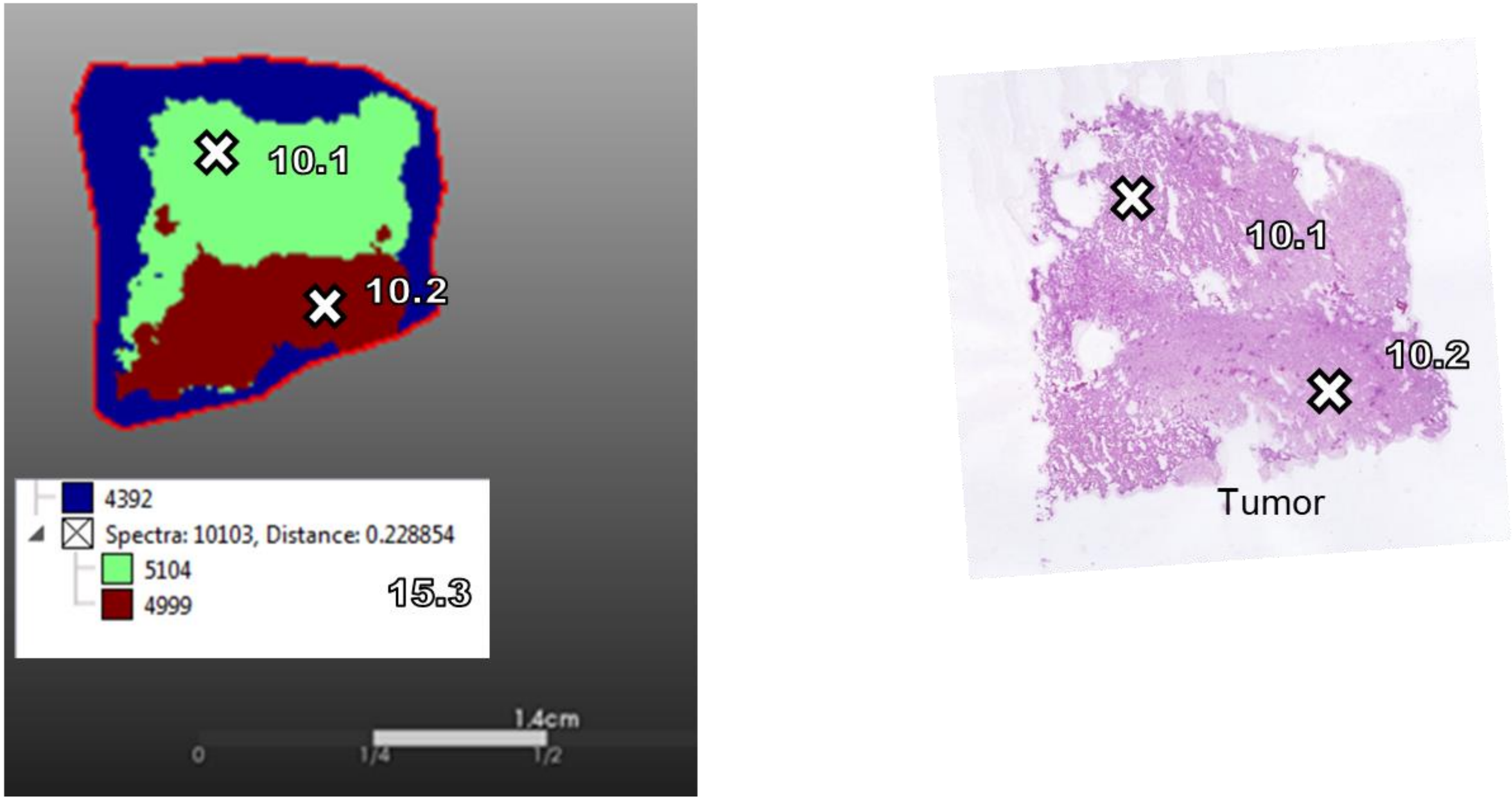
10.1
10.2
4392
Spectra: 10103, Distance: 0.228854
5104
4999
15.3
1.4cm
0
1/4
1/2
10.1
10.2
Tumor

9

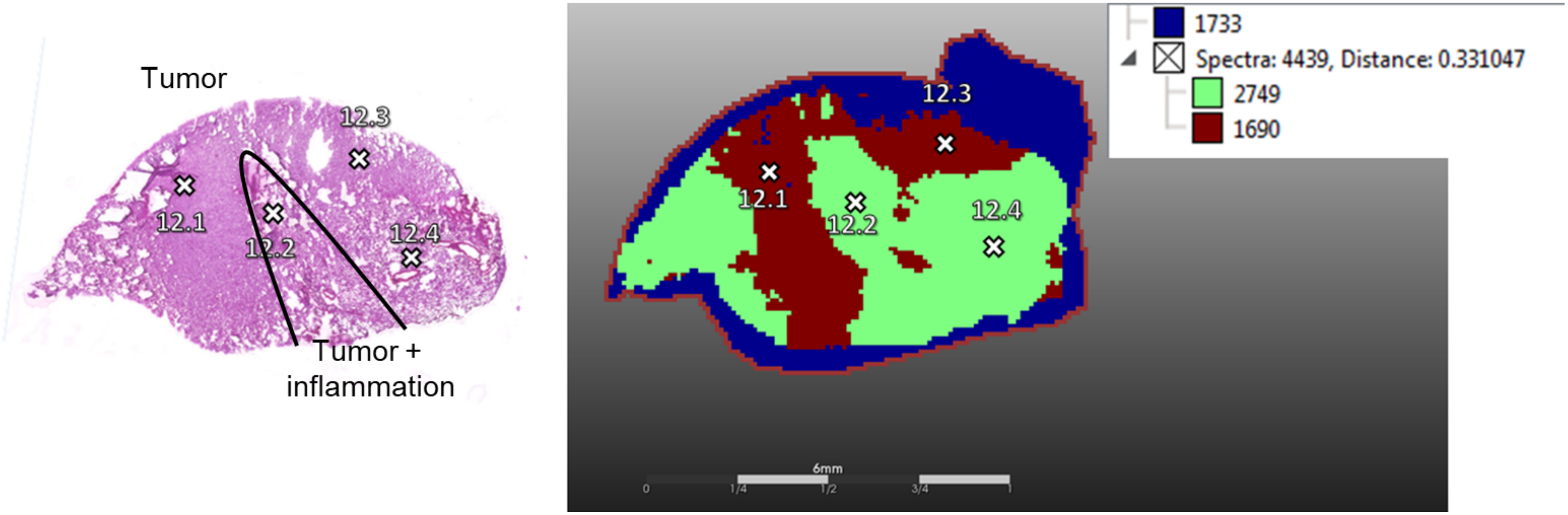
Tumor
12.3
12.1
12.2
12.4
Tumor + inflammation
1733
Spectra: 4439, Distance: 0.331047
2749
1690
12.3
12.1
12.2
12.4
6mm
0
1/4
1/2
3/4
1

10

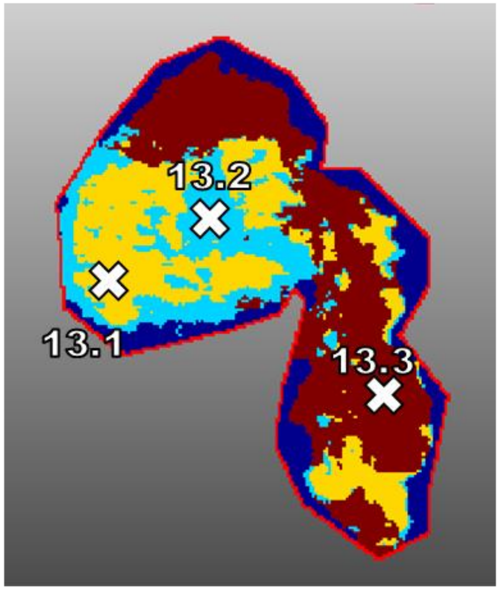

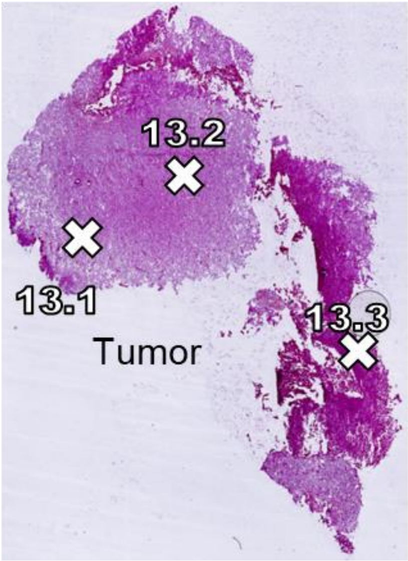

11

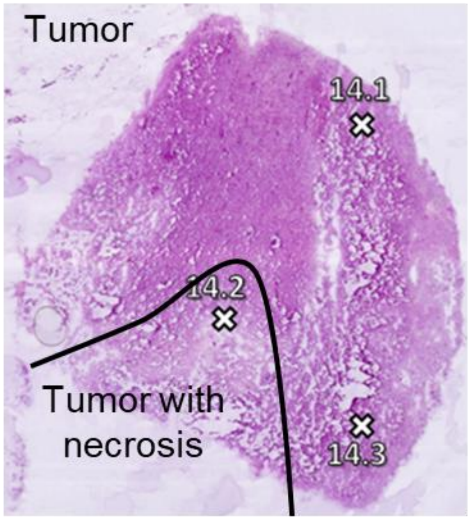
Tumor
14.1
14.2
Tumor with
necrosis
14.3

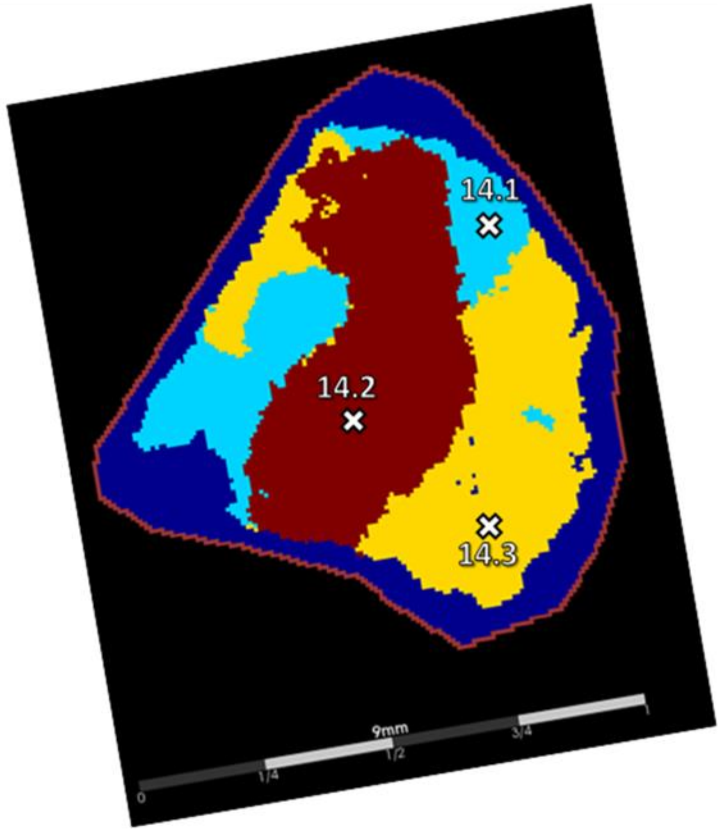
14.1
14.2
14.3
9mm
0
1/4
1/2
3/4
1

12

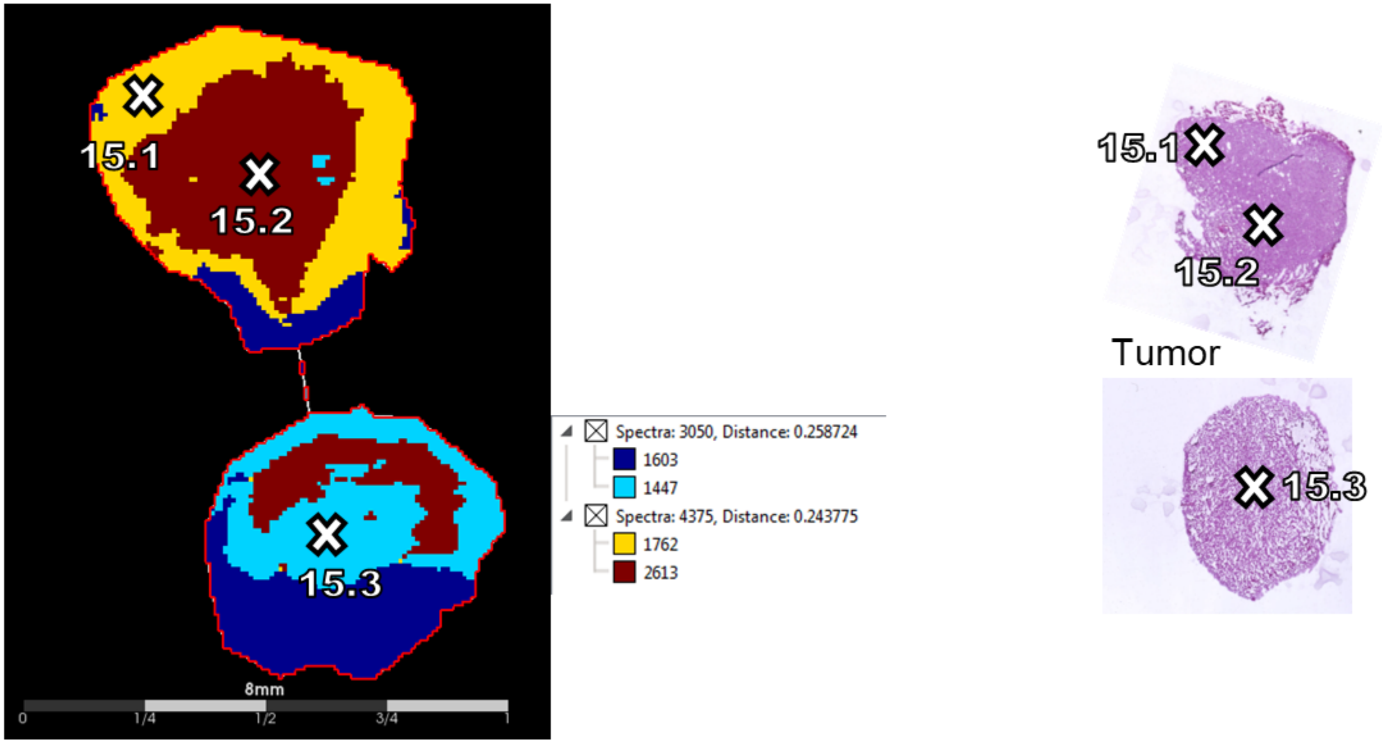
15.1
15.2
15.3
8mm
0
1/4
1/2
3/4
1
Spectra: 3050, Distance: 0.258724
1603
1447
Spectra: 4375, Distance: 0.243775
1762
2613
15.1
15.2
Tumor
15.3

13

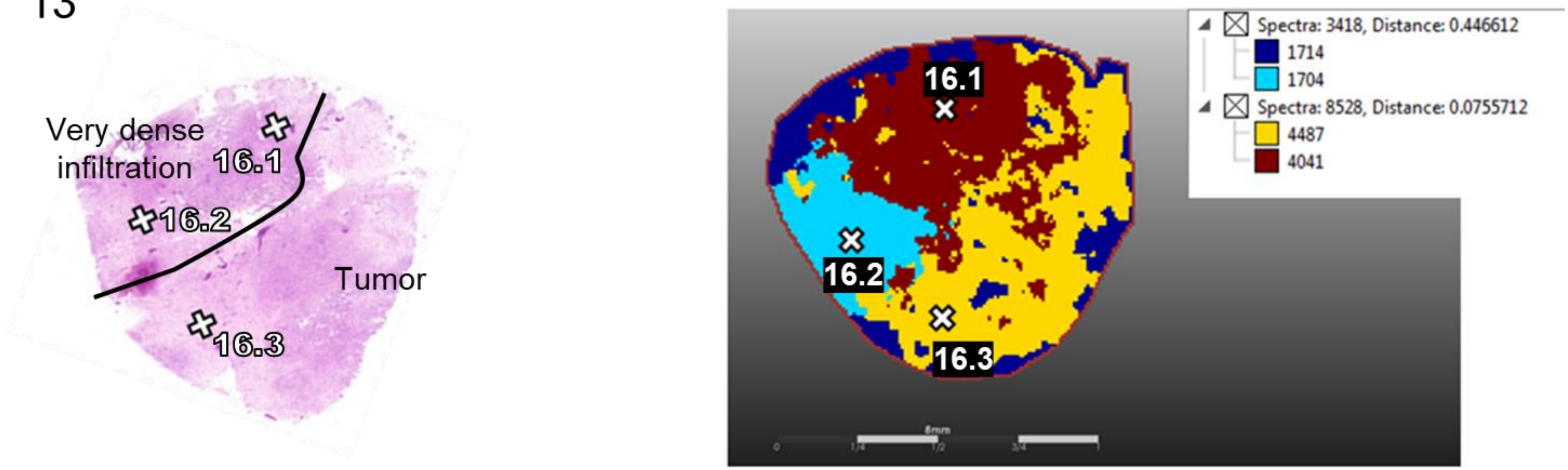

Very dense infiltration
16.1
16.2
16.3
Tumor
16.1
16.2
16.3
Spectra: 3418, Distance: 0.446612
1714
1704
Spectra: 8528, Distance: 0.0755712
4487
4041

14

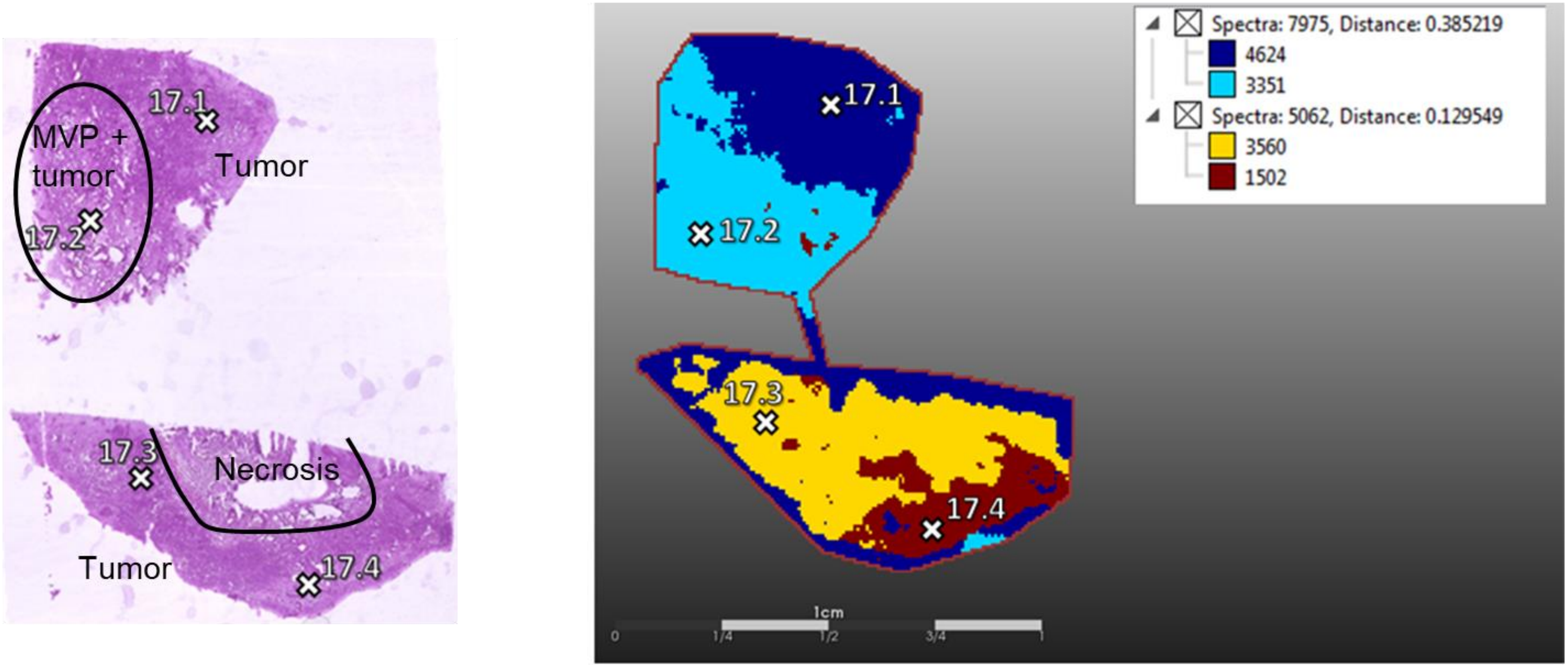
17.1
MVP +
tumor
Tumor
17.2
17.3
Necrosis
Tumor
17.4
Spectra: 7975, Distance: 0.385219
4624
3351
Spectra: 5062, Distance: 0.129549
3560
1502
17.1
17.2
17.3
17.4
1cm
0
1/4
1/2
3/4
1

15

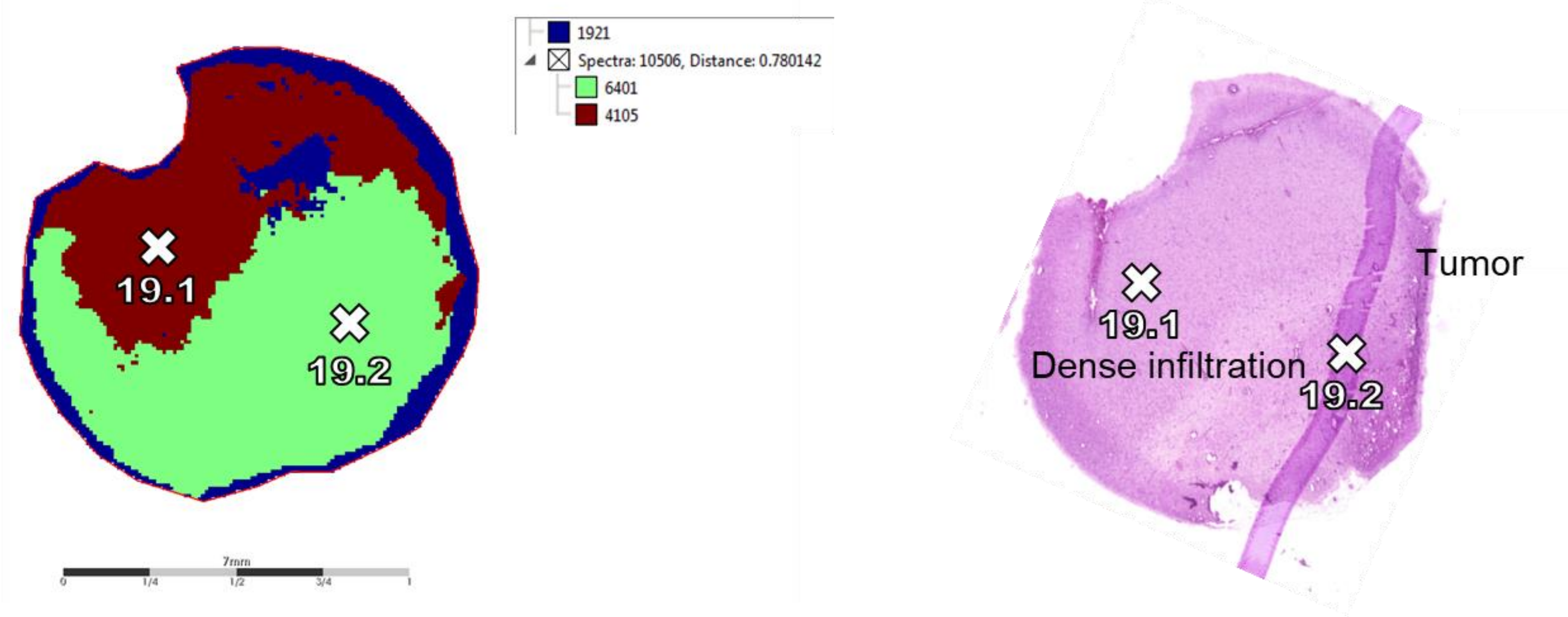
1921
Spectra: 10506, Distance: 0.780142
6401
4105
19.1
19.2
7mm
0
1/4
1/2
3/4
1
Tumor
19.1
Dense infiltration
19.2

16

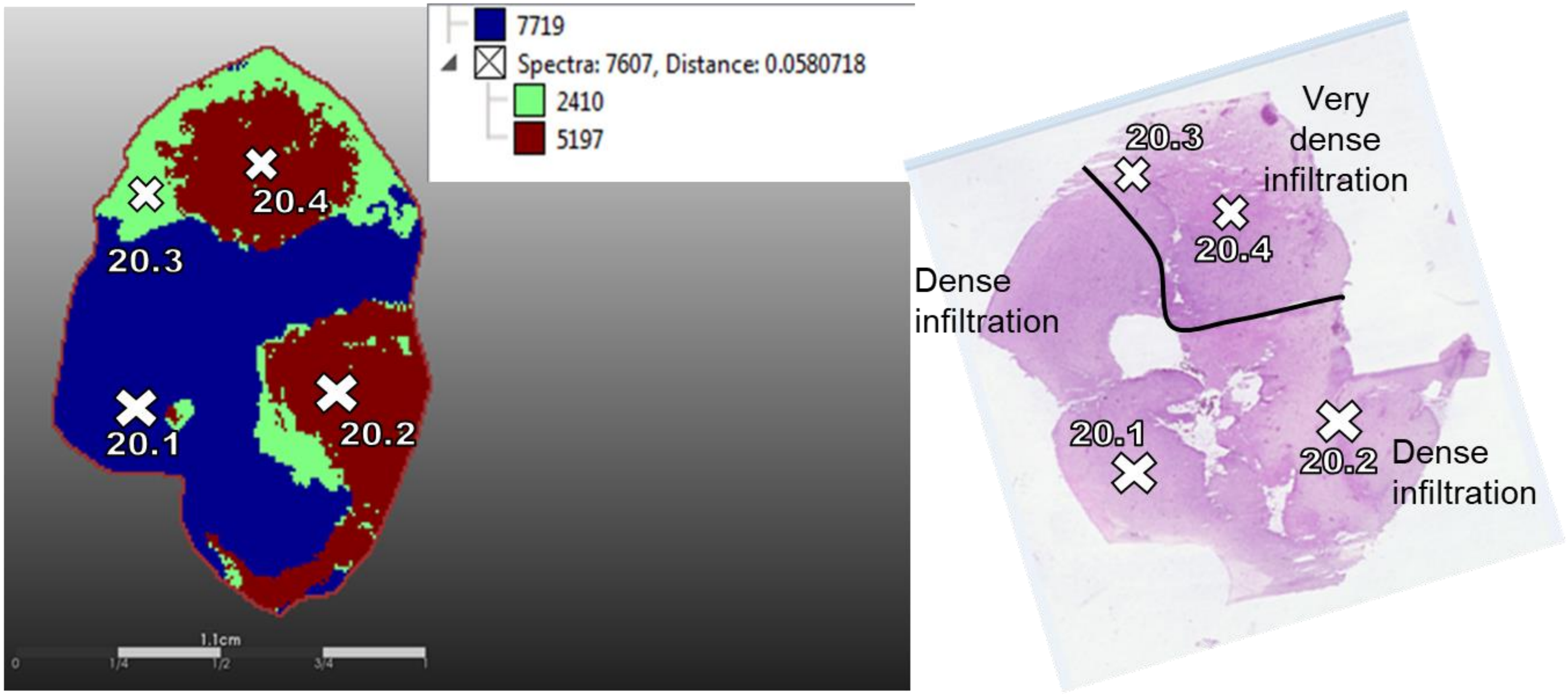
7719
Spectra: 7607, Distance: 0.0580718
2410
5197
20.4
20.3
20.1
20.2
1.1cm
0
1/4
1/2
3/4
1
Very dense infiltration
20.3
20.4
Dense infiltration
20.1
20.2
Dense infiltration

17

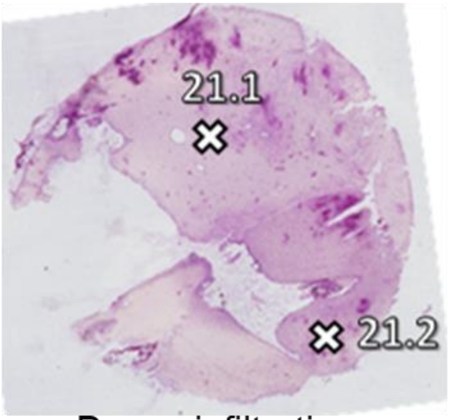

Dense infiltration

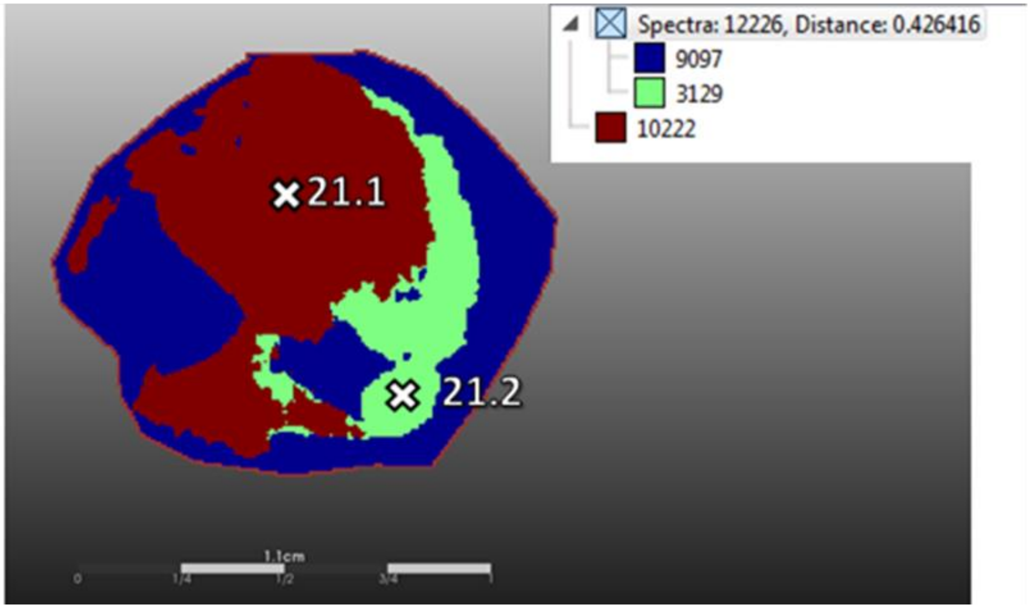

18

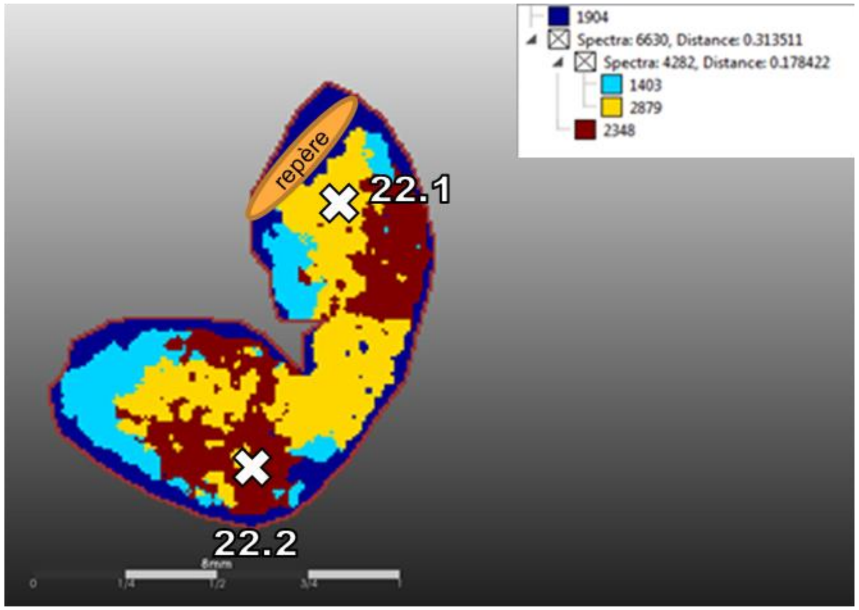
1904
Spectra: 6630, Distance: 0.313511
Spectra: 4282, Distance: 0.178422
1403
2879
2348
repère
22.1
22.2

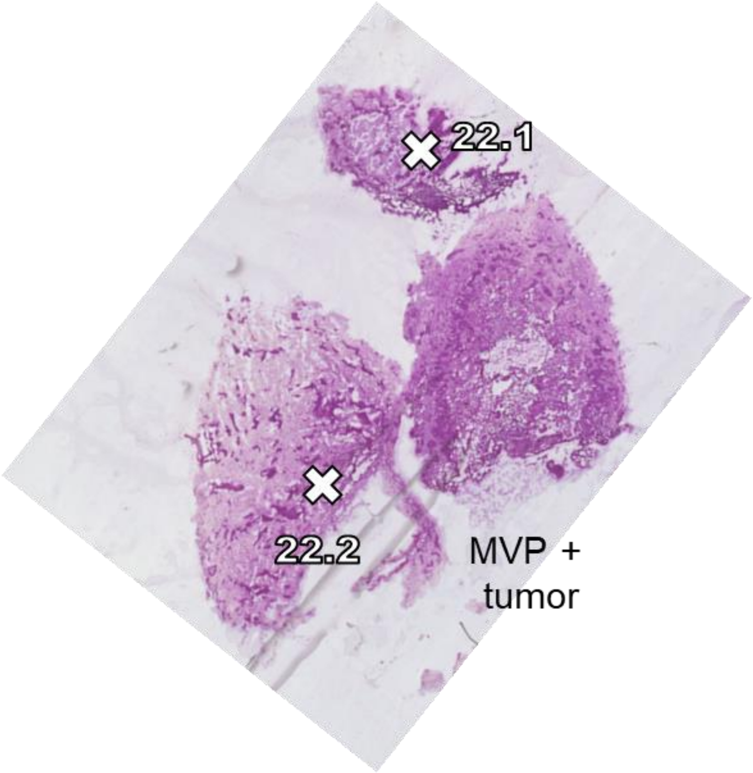
22.1
22.2
MVP +
tumor

19

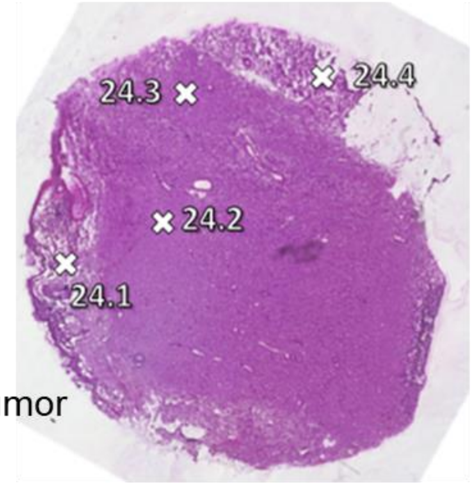
24.3
24.4
24.2
24.1

Tumor

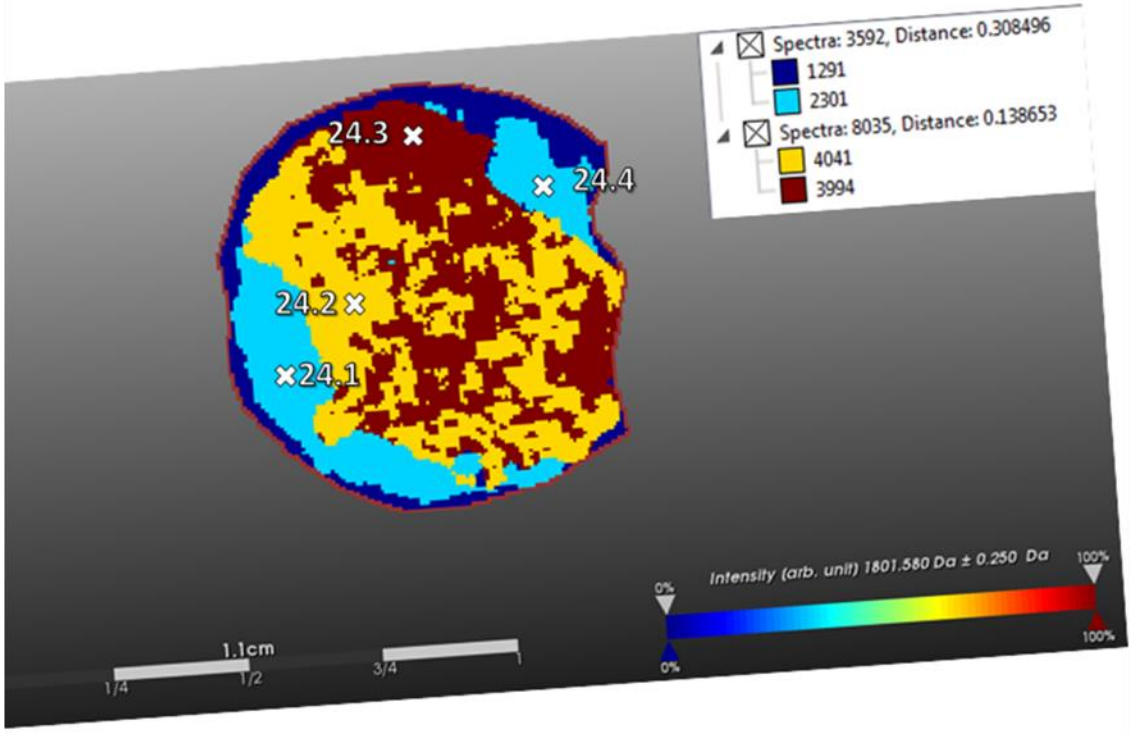
Spectra: 3592, Distance: 0.308496
1291
2301
Spectra: 8035, Distance: 0.138653
4041
3994
24.3
24.4
24.2
24.1
Intensity (arb. unit) 1801.580 Da ± 0.250 Da
0%
100%
1.1cm
1/4
1/2
3/4

20

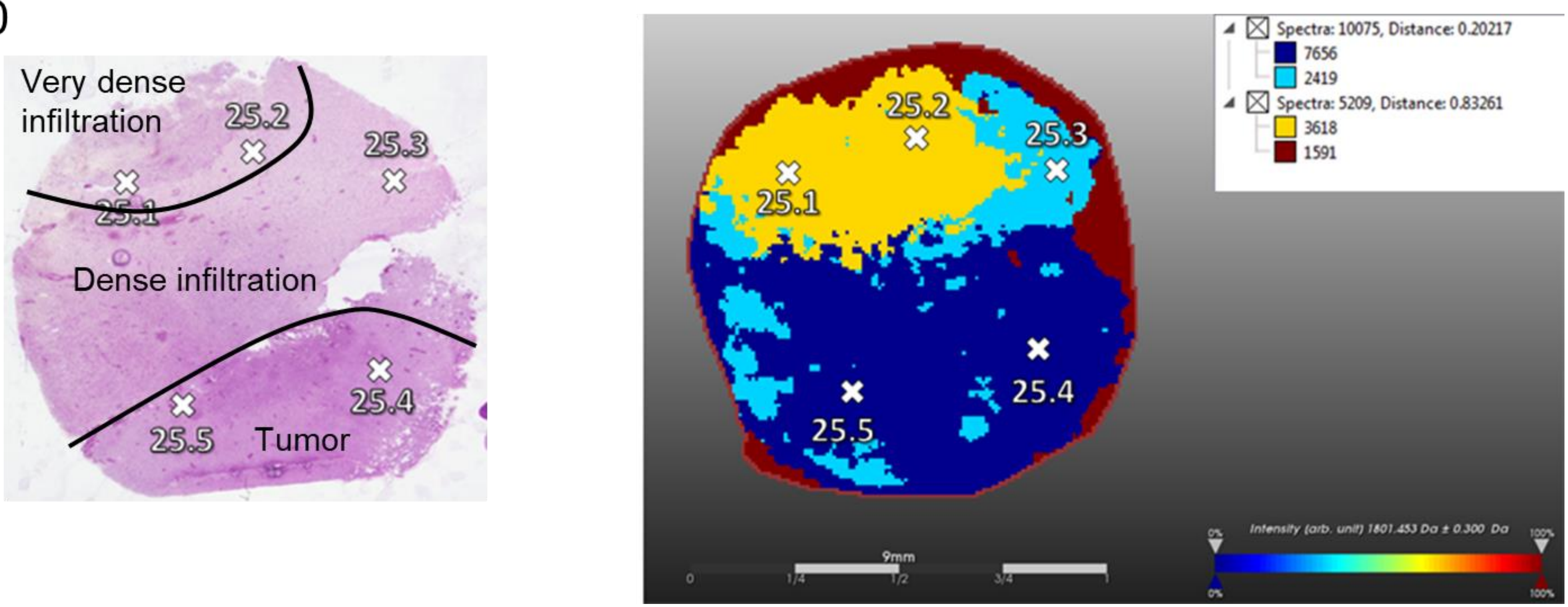

Very dense infiltration
25.2
25.3
25.1
Dense infiltration
25.4
25.5
Tumor
Spectra: 10075, Distance: 0.20217
7656
2419
Spectra: 5209, Distance: 0.83261
3618
1591
25.2
25.3
25.1
25.4
25.5
Intensity (arb. unit) 1801.453 Da ± 0.300 Da
9mm
0%
100%

21

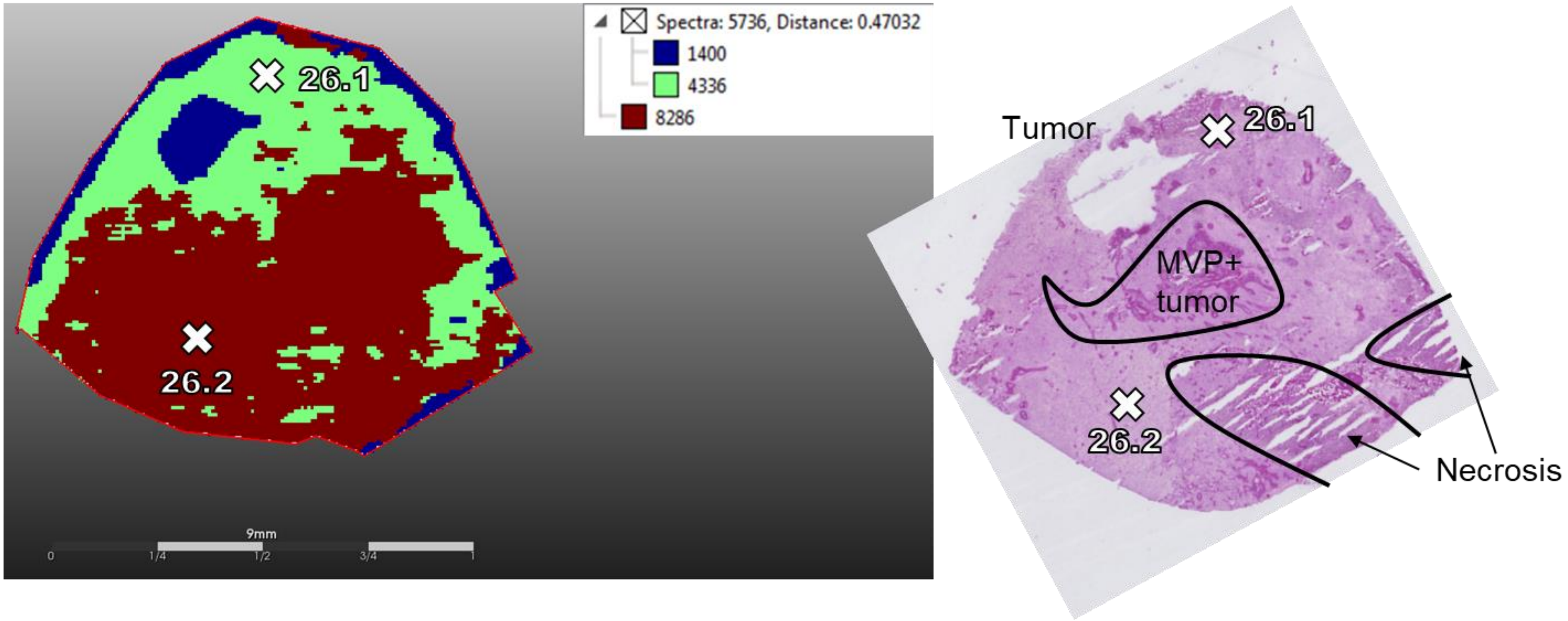

Spectra: 5736, Distance: 0.47032
1400
4336
8286
26.1
26.2
9mm
0
1/4
1/2
3/4
1
Tumor
MVP+
tumor
Necrosis

22

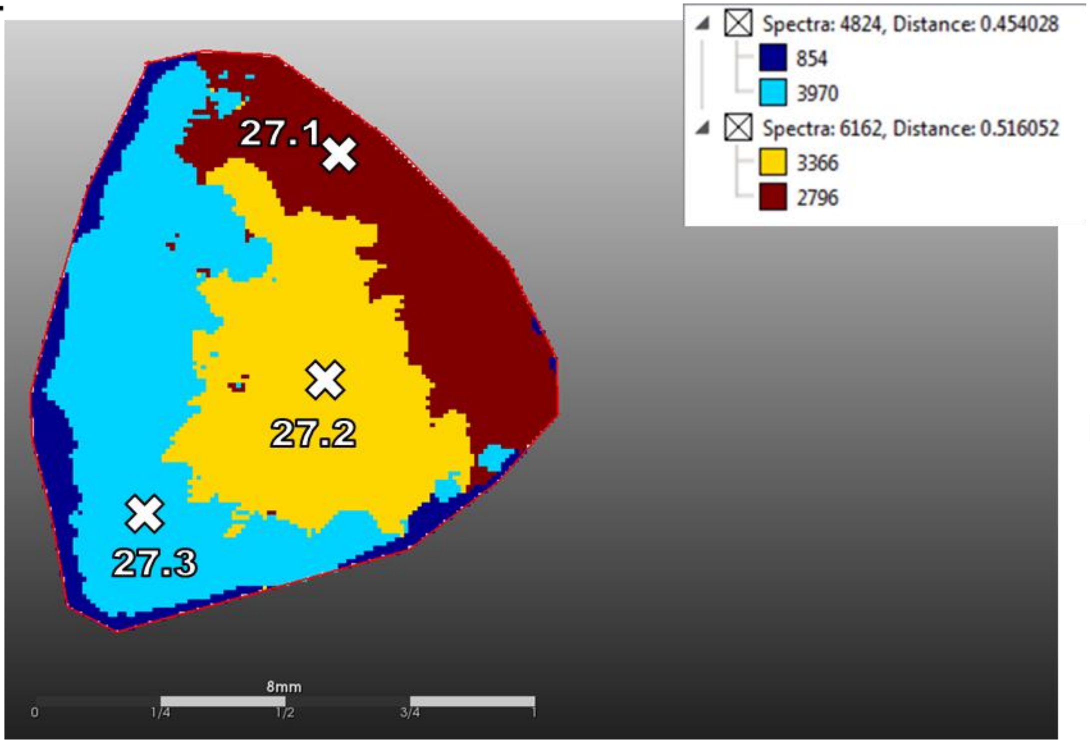

Spectra: 4824, Distance: 0.454028
854
3970
Spectra: 6162, Distance: 0.516052
3366
2796
27.1
27.2
27.3
8mm
0
1/4
1/2
3/4
1

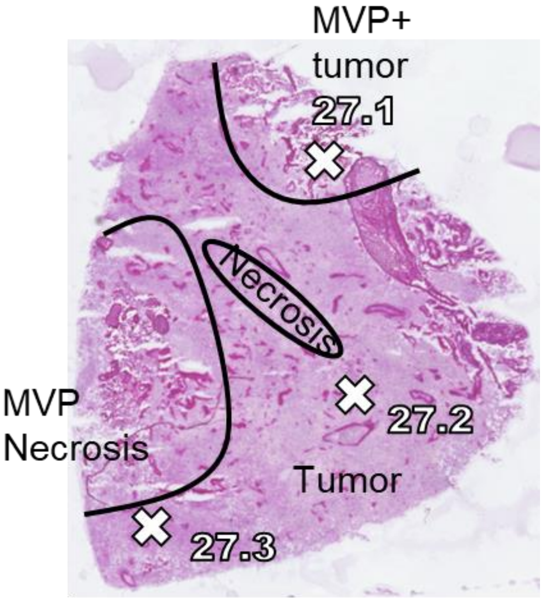

MVP+
tumor
27.1
Necrosis
MVP
Necrosis
27.2
Tumor
27.3

23

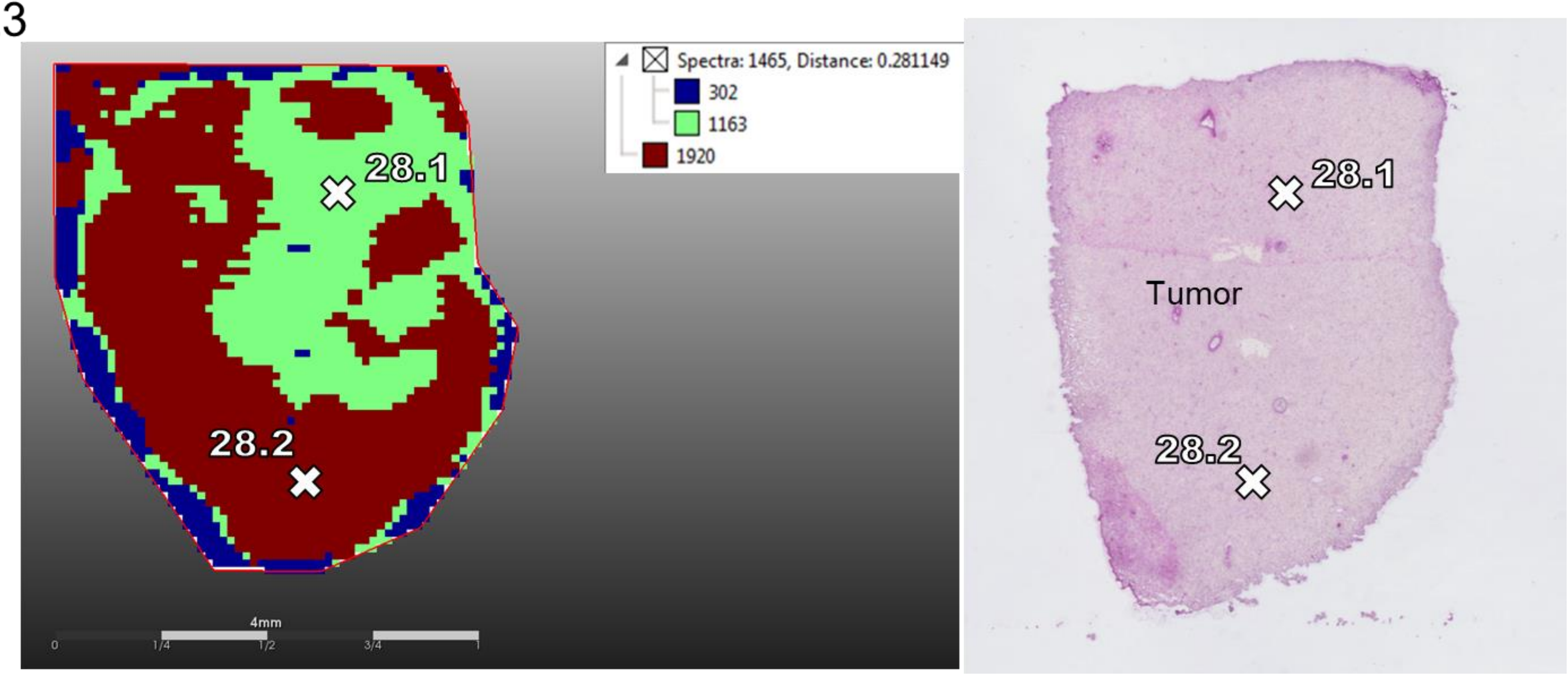
Spectra: 1465, Distance: 0.281149
302
1163
1920
28.1
28.2
4mm
0
1/4
1/2
3/4
1
Tumor
28.1
28.2

24

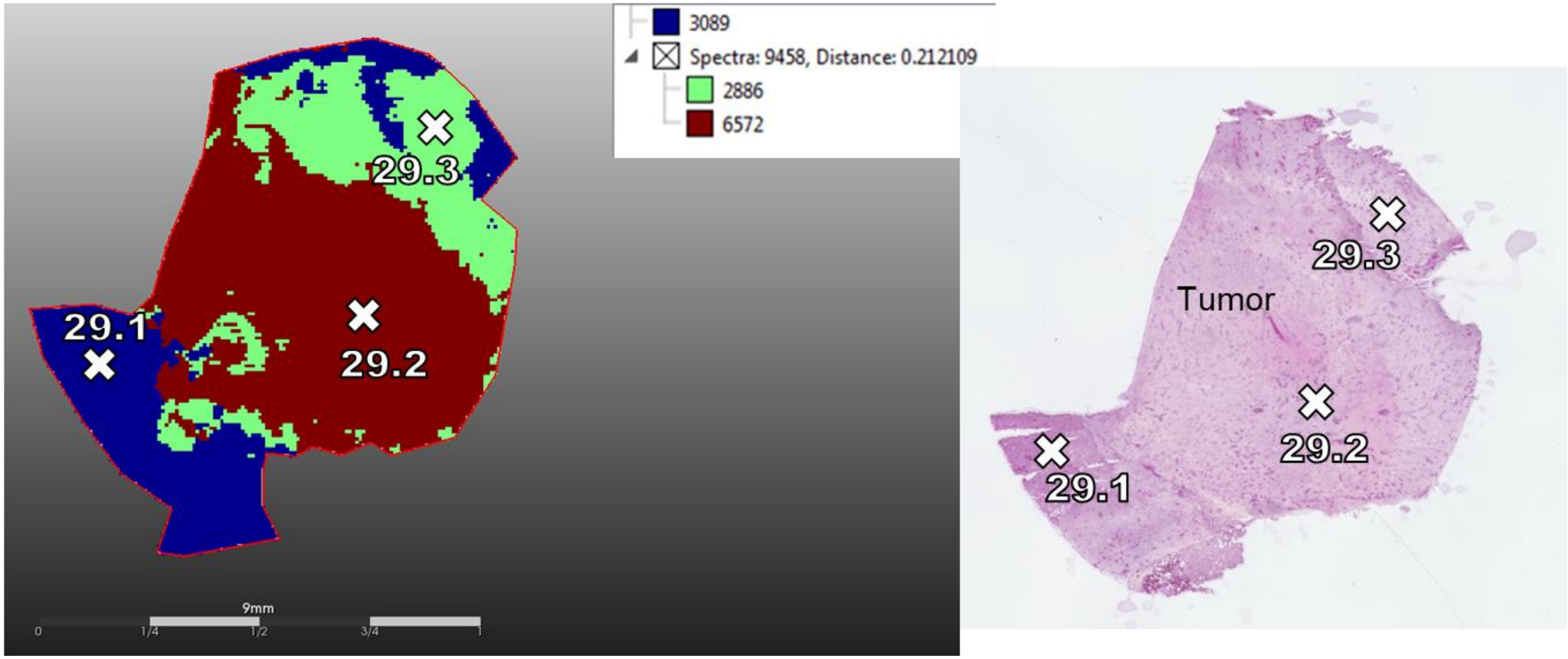
3089
Spectra: 9458, Distance: 0.212109
2886
6572
29.3
29.1
29.2
9mm
0
1/4
1/2
3/4
1
Tumor
29.3
29.2
29.1

25

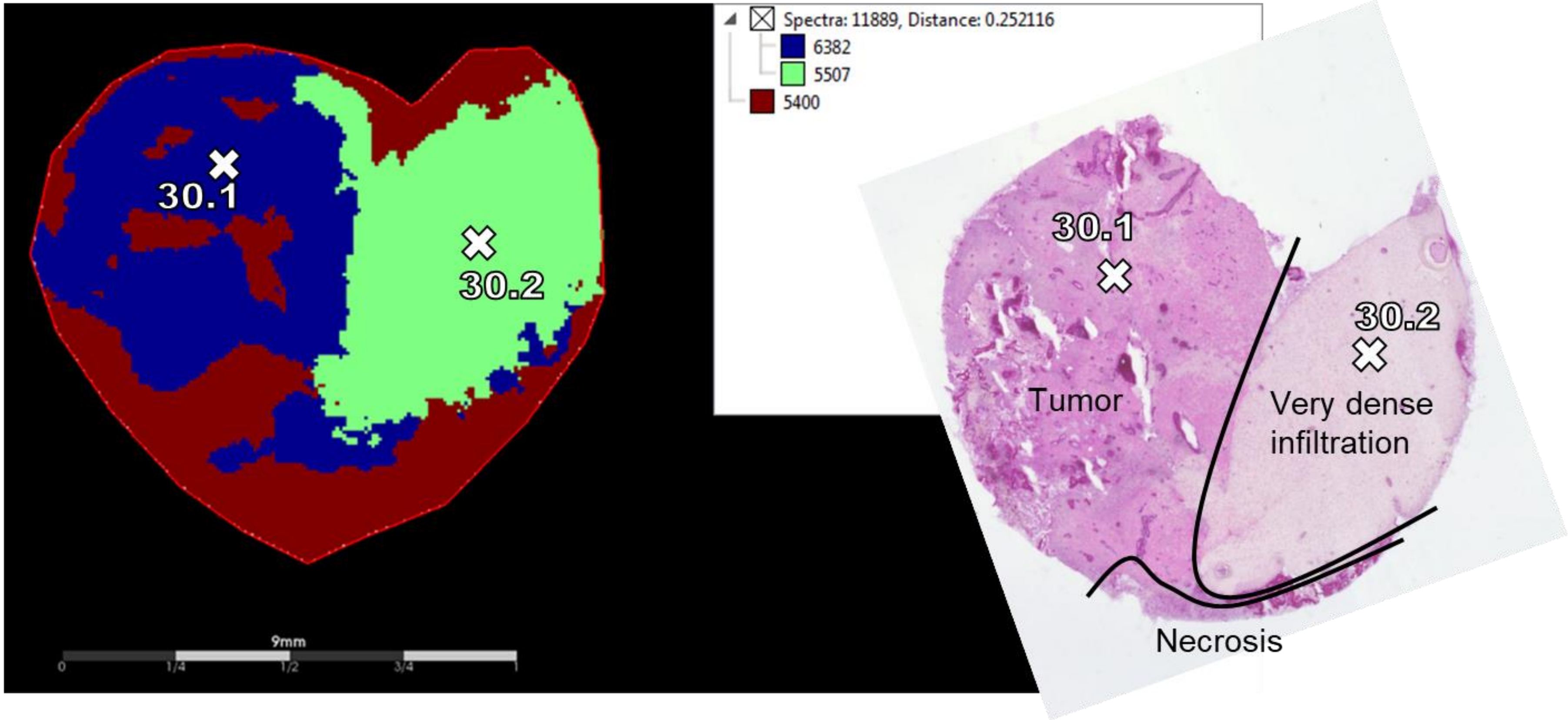
Spectra: 11889, Distance: 0.252116
6382
5507
5400
30.1
30.2
9mm
0
1/4
1/2
3/4
1
30.1
30.2
Tumor
Very dense
infiltration
Necrosis

26

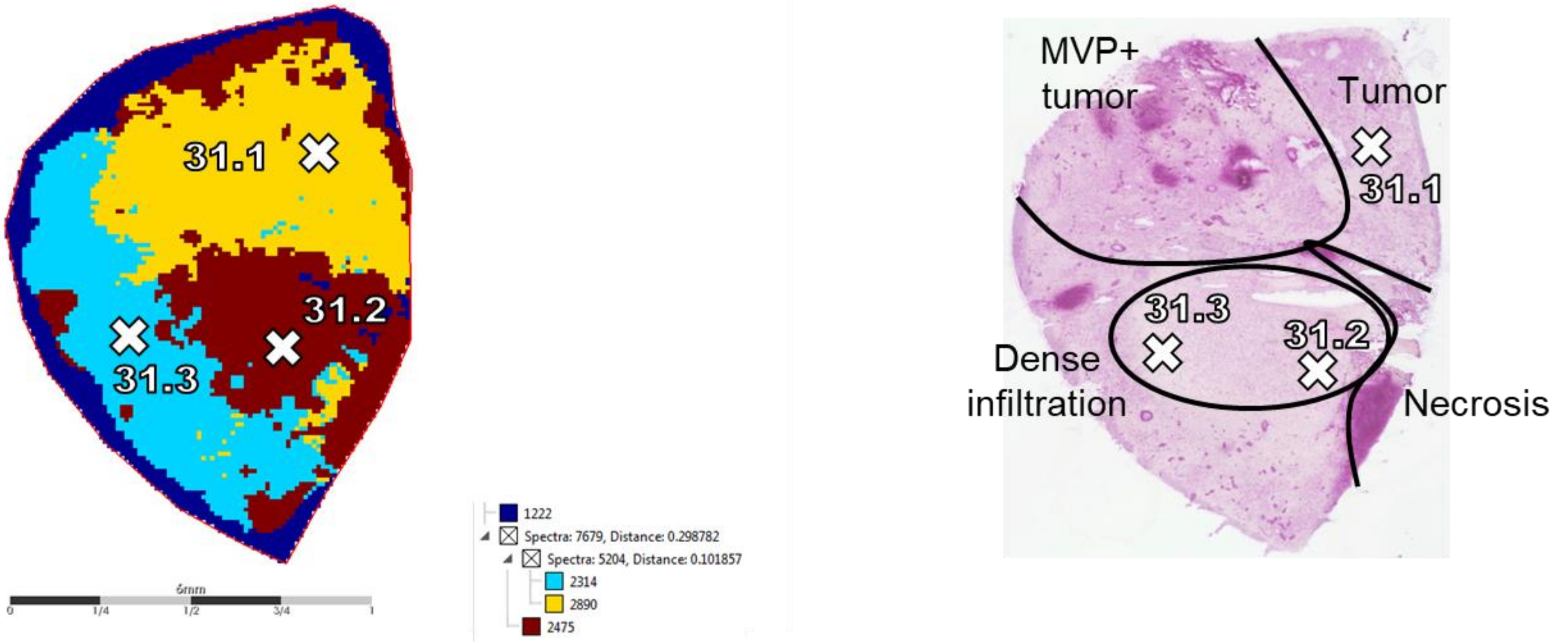

31.1
31.2
31.3
6mm
0
1/4
1/2
3/4
1
1222
Spectra: 7679, Distance: 0.298782
Spectra: 5204, Distance: 0.101857
2314
2890
2475
MVP+
tumor
Tumor
31.1
31.3
31.2
Dense
infiltration
Necrosis

27

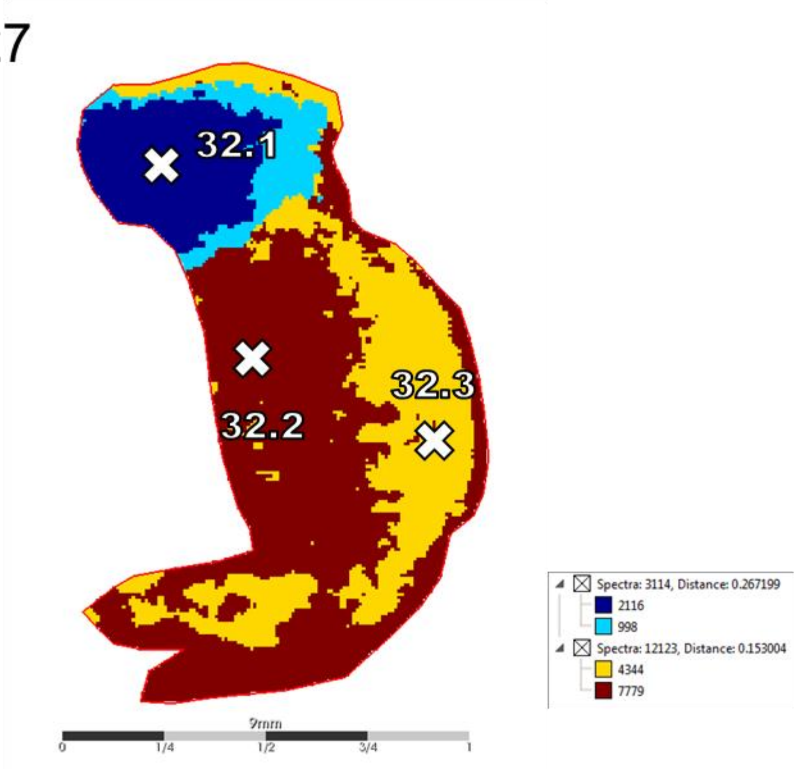
32.1
32.2
32.3
Spectra: 3114, Distance: 0.267199
2116
998
Spectra: 12123, Distance: 0.153004
4344
7779
9mm
0
1/4
1/2
3/4
1

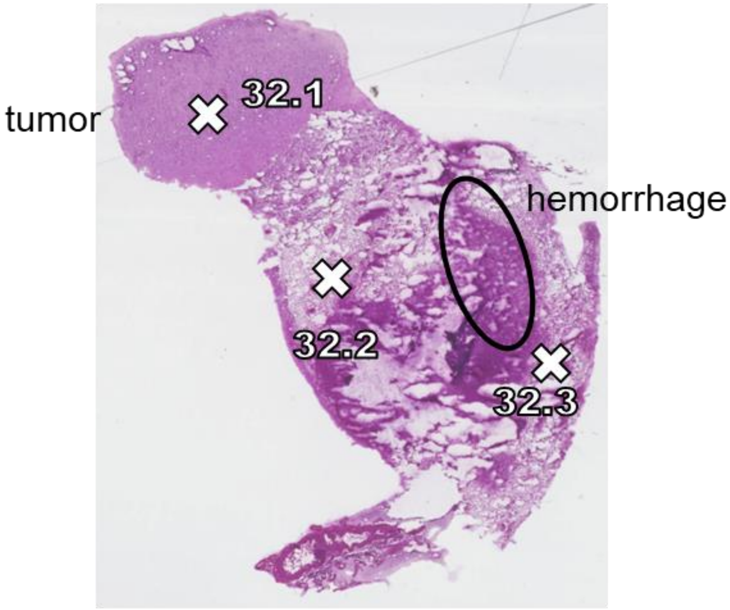
tumor
32.1
hemorrhage
32.2
32.3

28

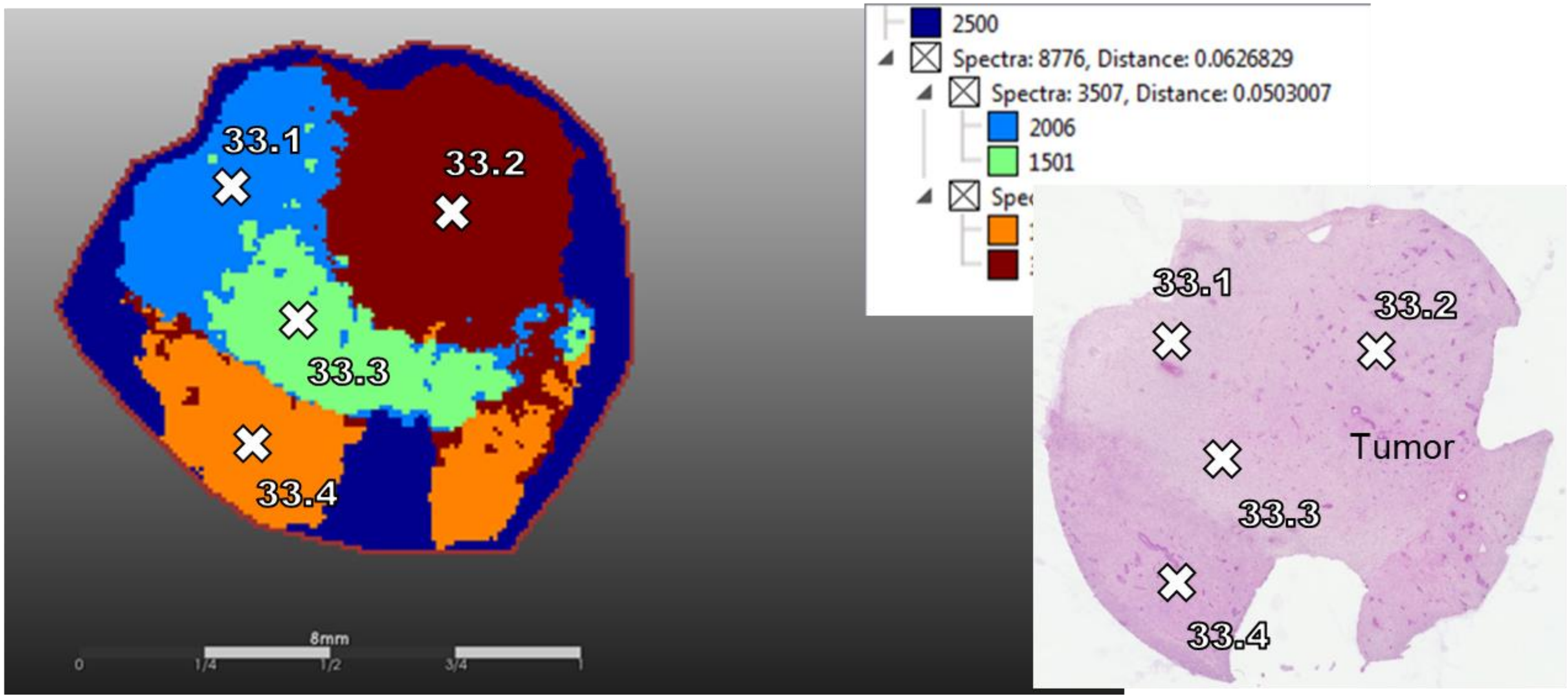
33.1
33.2
33.3
33.4
8mm
0
1/4
1/2
3/4
1
2500
Spectra: 8776, Distance: 0.0626829
Spectra: 3507, Distance: 0.0503007
2006
1501
Tumor

29

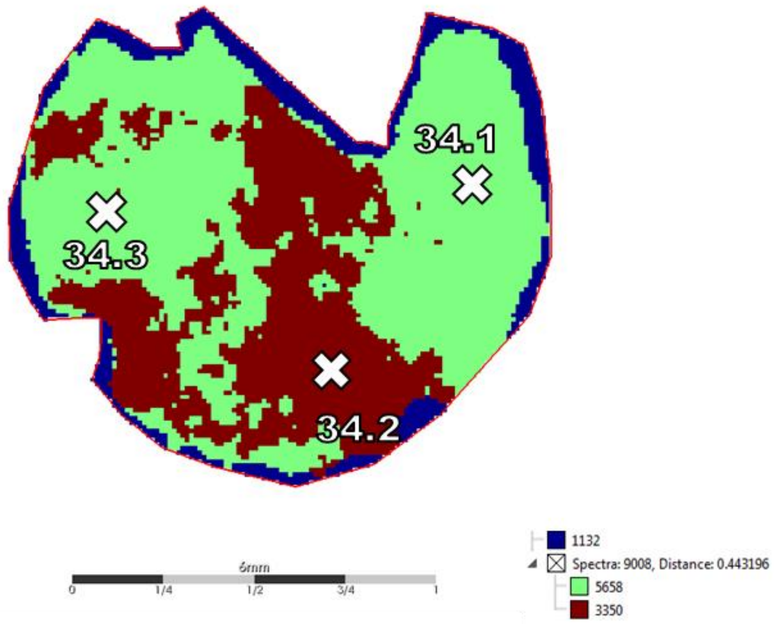
34.1
34.3
34.2
6mm
0
1/4
1/2
3/4
1
1132
Spectra: 9008, Distance: 0.443196
5658
3350

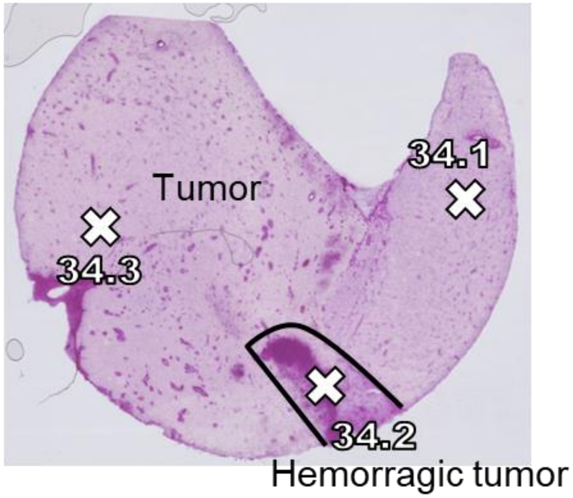
Tumor
34.1
34.3
34.2
Hemorragic tumor

30

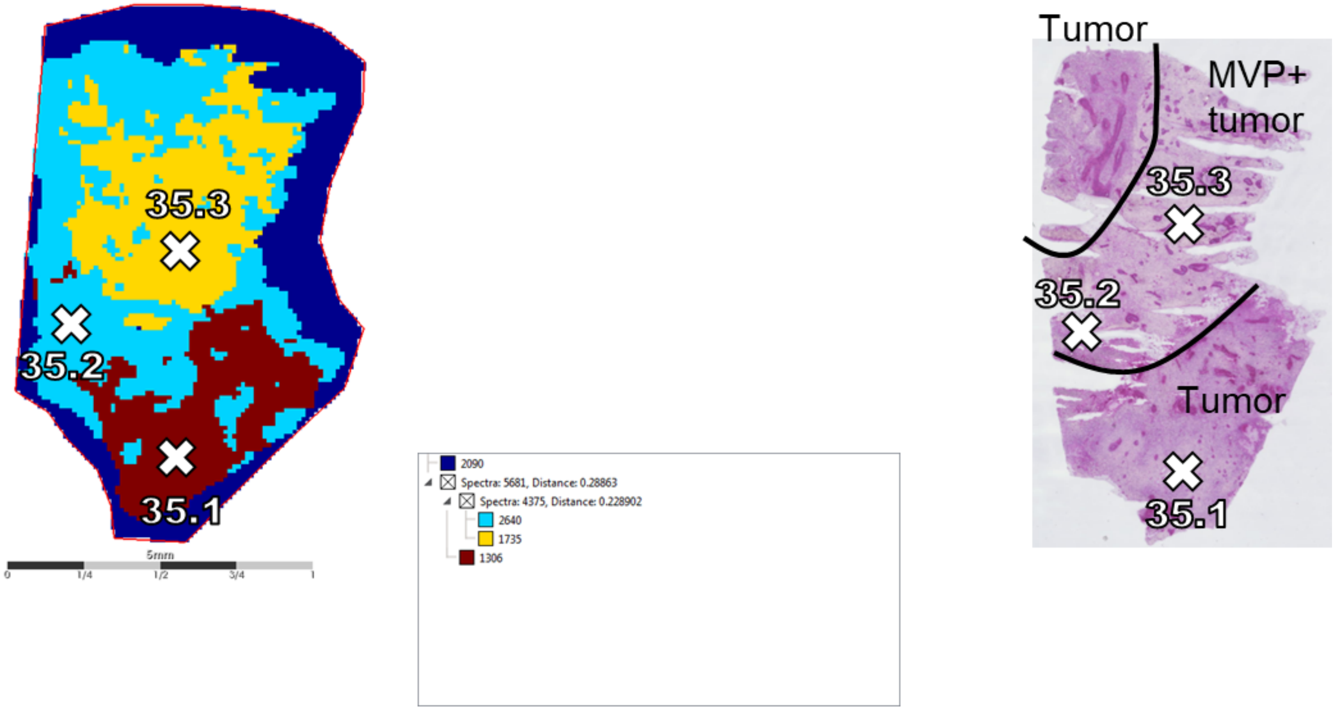
35.3
35.2
35.1
5mm
0
1/4
1/2
3/4
1
2090
Spectra: 5681, Distance: 0.28863
Spectra: 4375, Distance: 0.228902
2640
1735
1306
Tumor
MVP+ tumor
35.3
35.2
Tumor
35.1

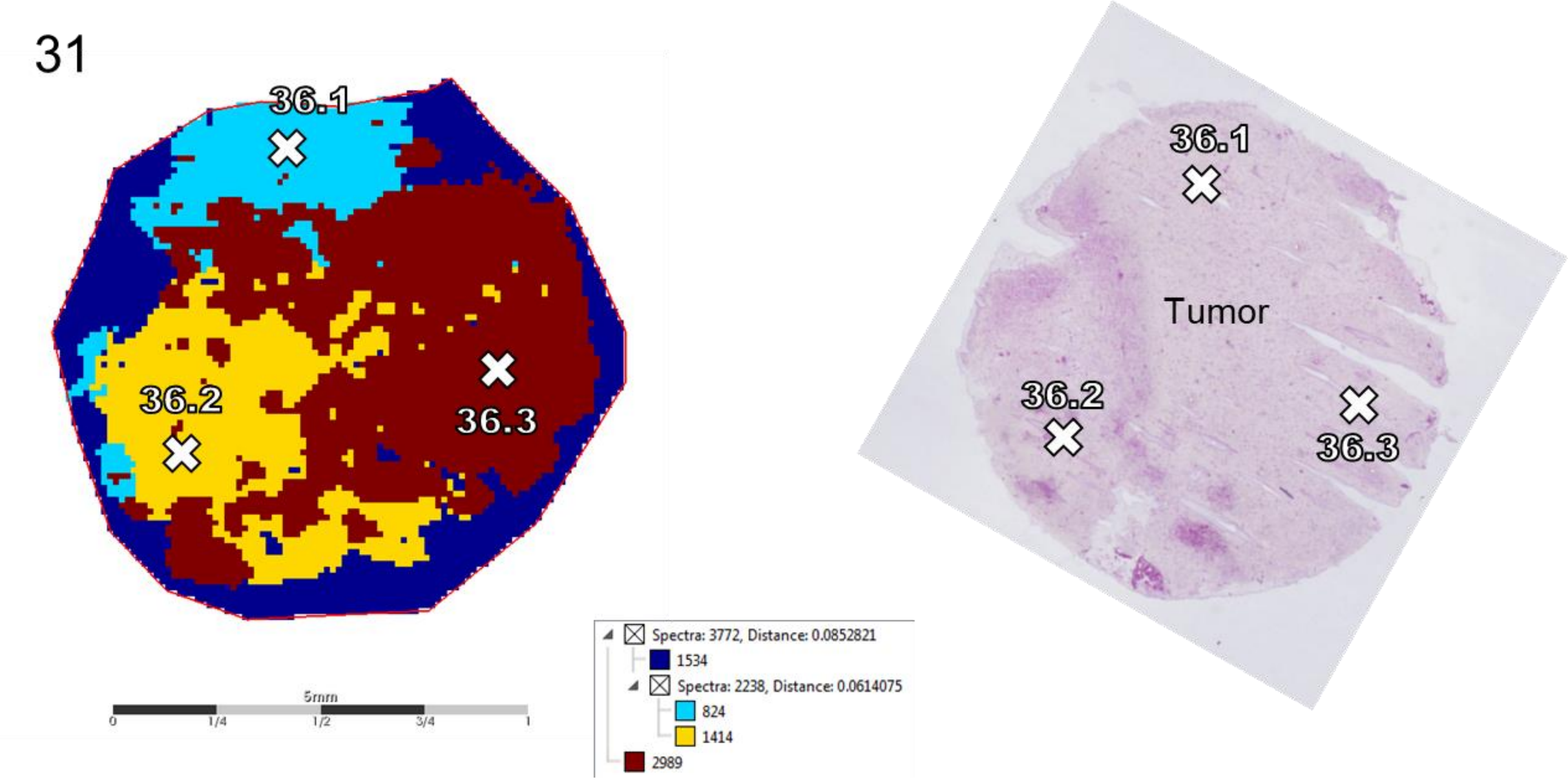
31
36.1
36.2
36.3
5mm
0
1/4
1/2
3/4
1
Spectra: 3772, Distance: 0.0852821
1534
Spectra: 2238, Distance: 0.0614075
824
1414
2989
Tumor

32

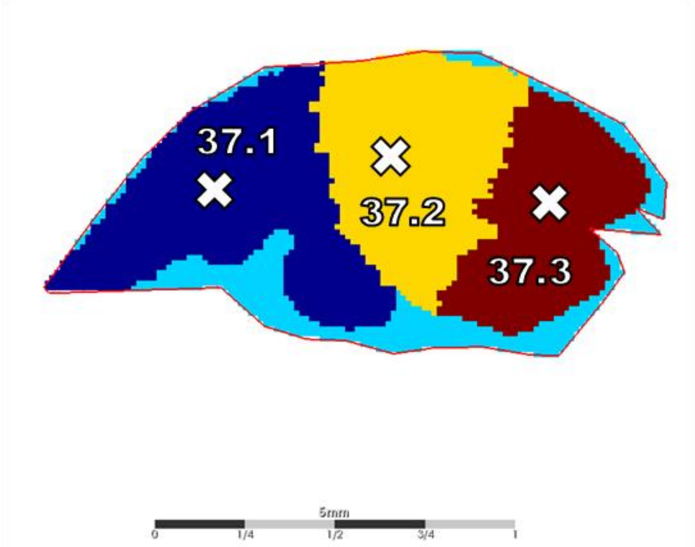
37.1
37.2
37.3
5mm
0
1/4
1/2
3/4
1

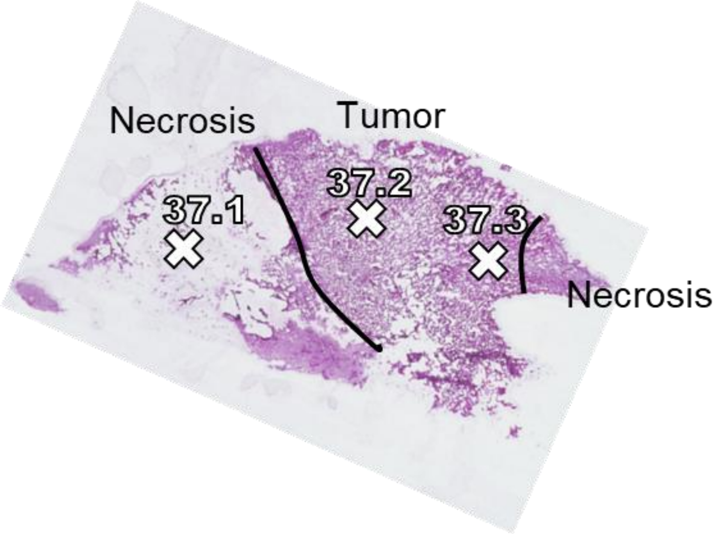
Necrosis
Tumor
37.1
37.2
37.3
Necrosis

33

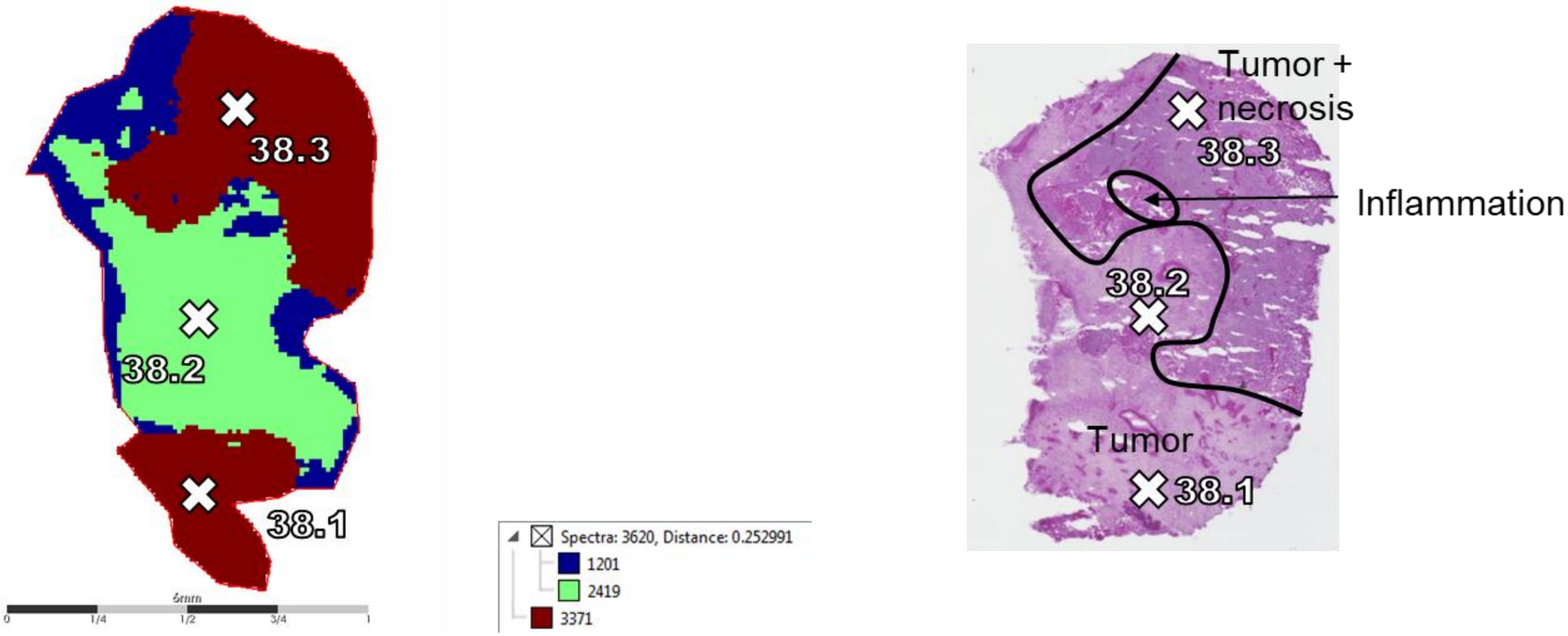
38.3
38.2
38.1
Spectra: 3620, Distance: 0.252991
1201
2419
3371
Tumor + necrosis
38.3
Inflammation
38.2
Tumor
38.1

34

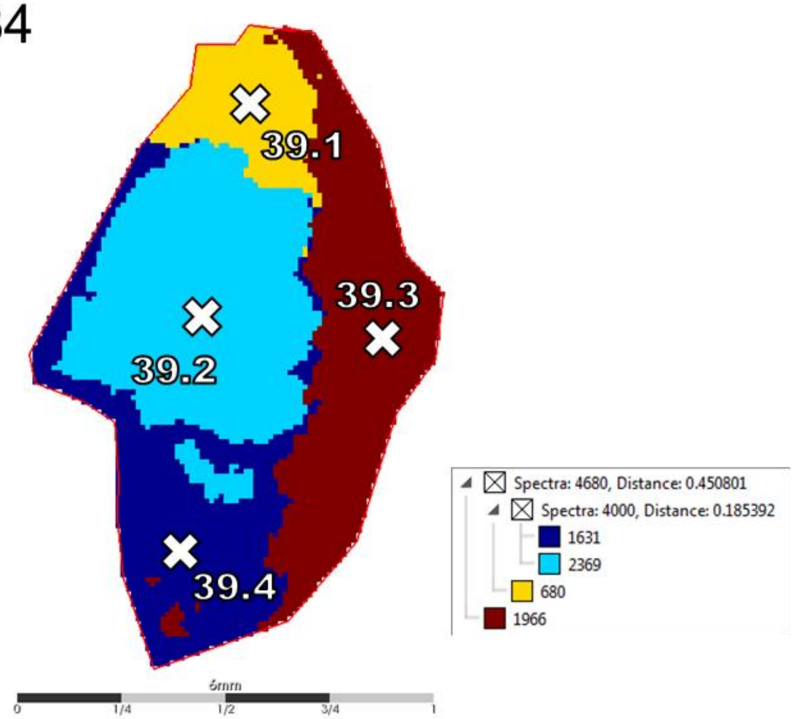
39.1
39.3
39.2
39.4
Spectra: 4680, Distance: 0.450801
Spectra: 4000, Distance: 0.185392
1631
2369
680
1966
6mm
0
1/4
1/2
3/4
1

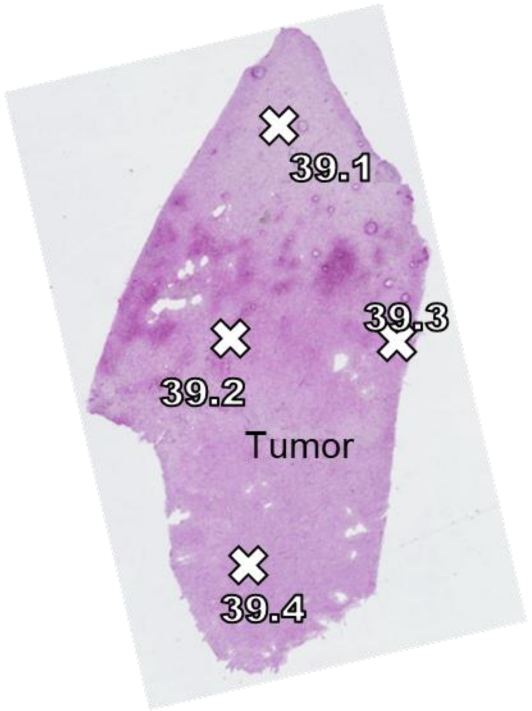
39.1
39.3
39.2
Tumor
39.4

35

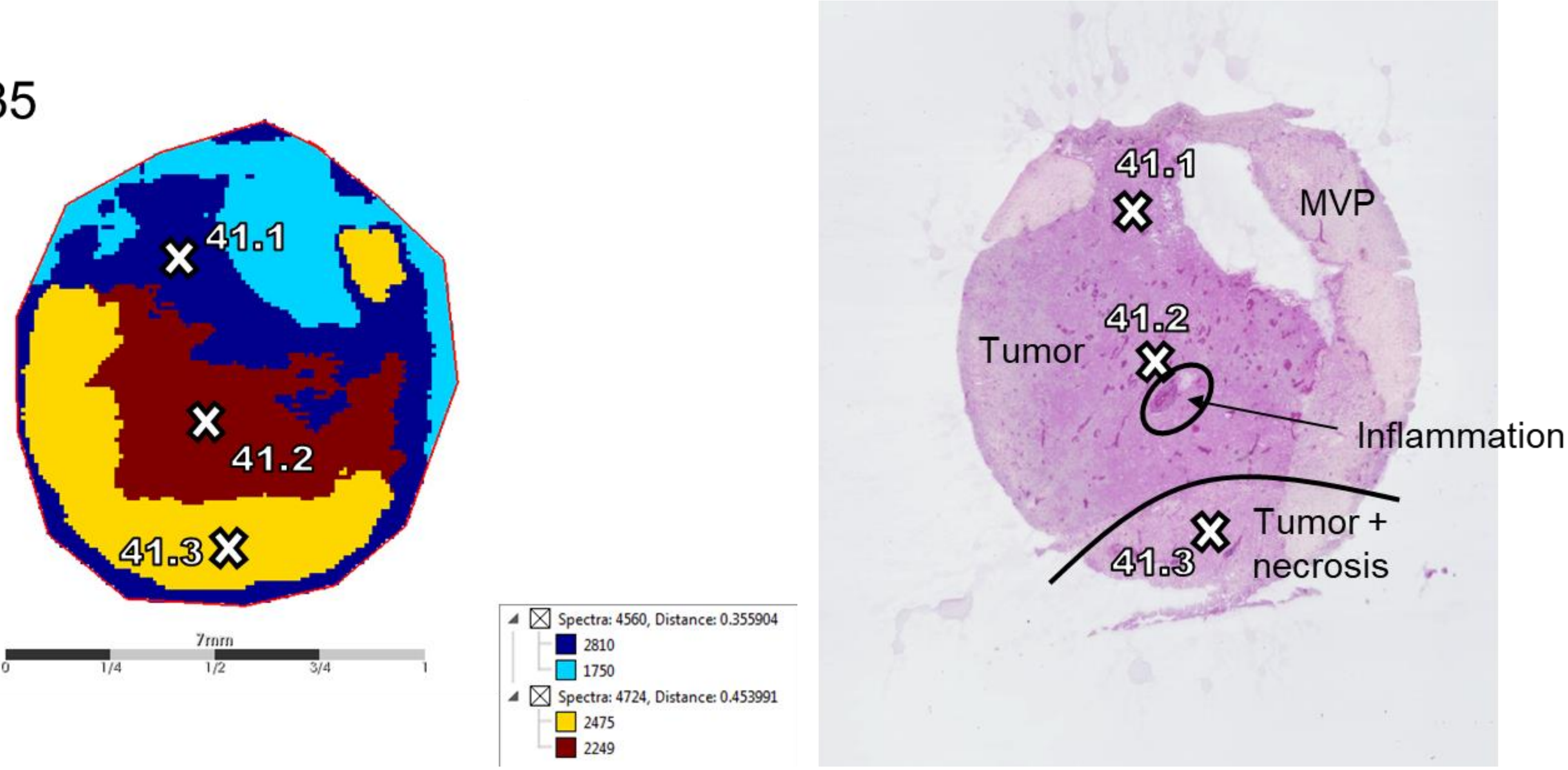
41.1
41.2
41.3
7mm
0
1/4
1/2
3/4
1
Spectra: 4560, Distance: 0.355904
2810
1750
Spectra: 4724, Distance: 0.453991
2475
2249
41.1
MVP
41.2
Tumor
Inflammation
Tumor +
necrosis
41.3

36

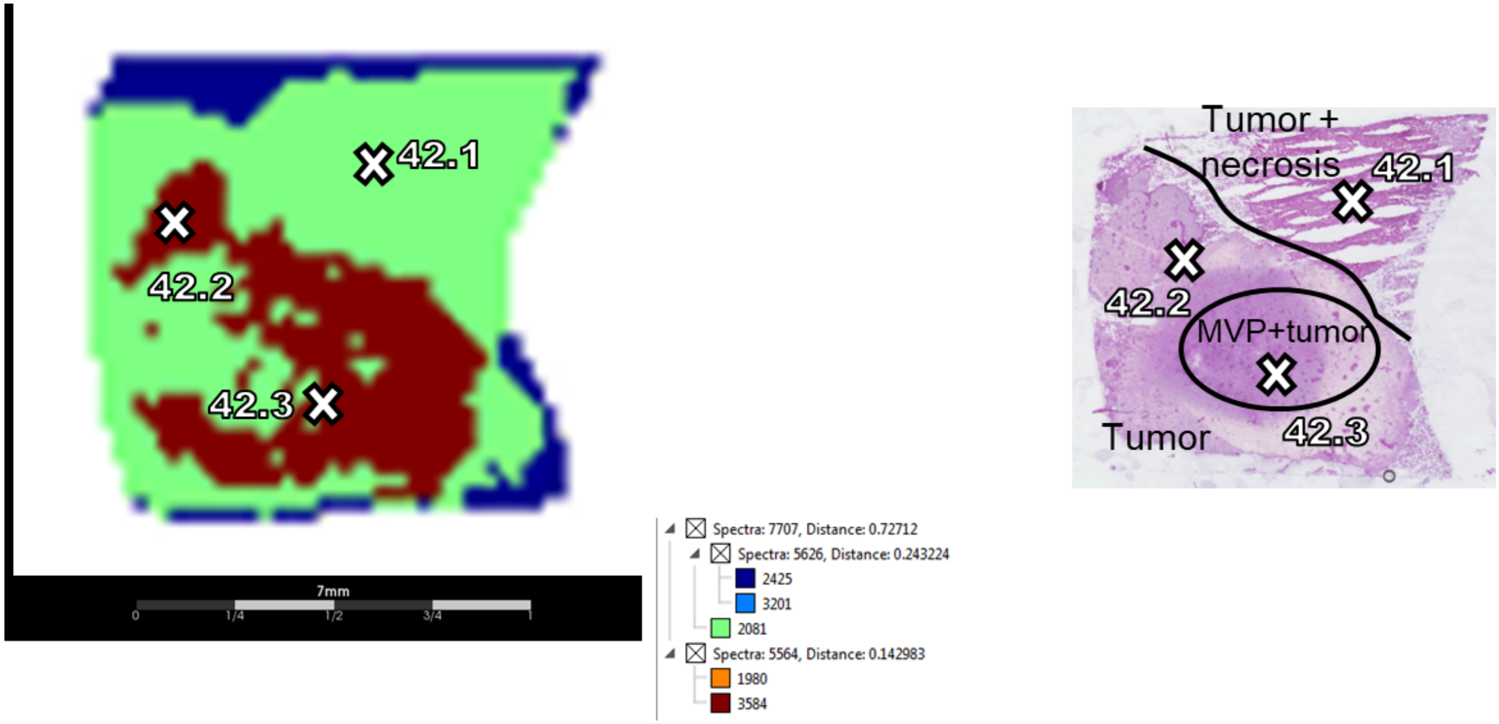
42.1
42.2
42.3
7mm
0
1/4
1/2
3/4
1
Spectra: 7707, Distance: 0.72712
Spectra: 5626, Distance: 0.243224
2425
3201
2081
Spectra: 5564, Distance: 0.142983
1980
3584
Tumor +
necrosis
42.1
42.2
MVP+tumor
Tumor
42.3

37

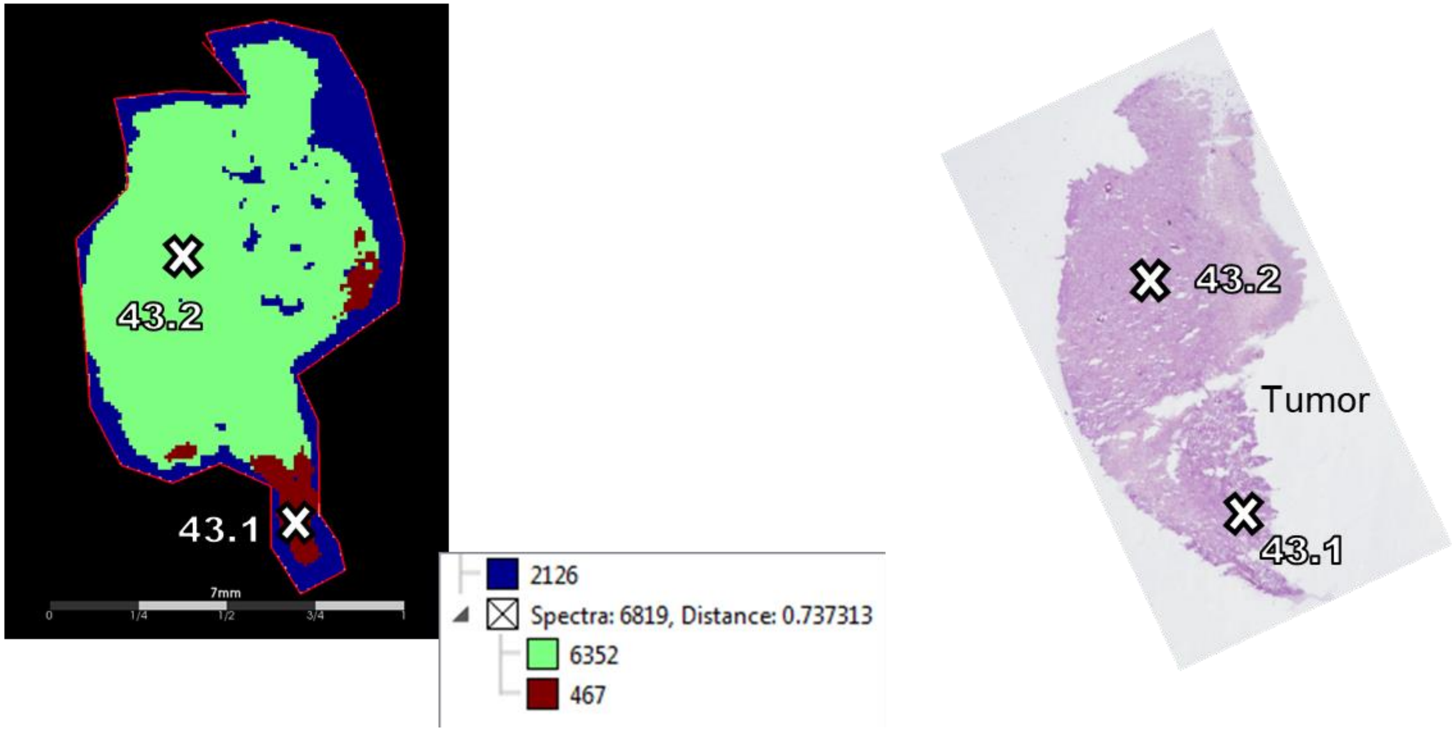
43.2
43.1
7mm
0
1/4
1/2
3/4
1
2126
Spectra: 6819, Distance: 0.737313
6352
467
43.2
Tumor
43.1

38

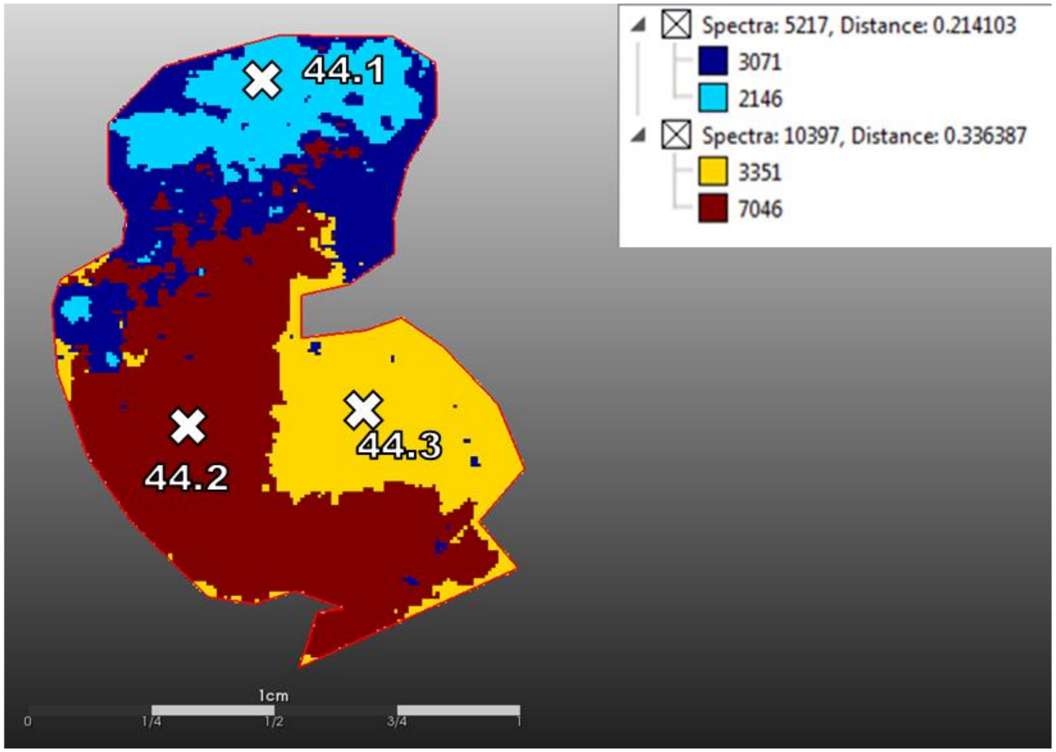

44.1
44.2
44.3
Spectra: 5217, Distance: 0.214103
3071
2146
Spectra: 10397, Distance: 0.336387
3351
7046
1cm
0
1/4
1/2
3/4
1

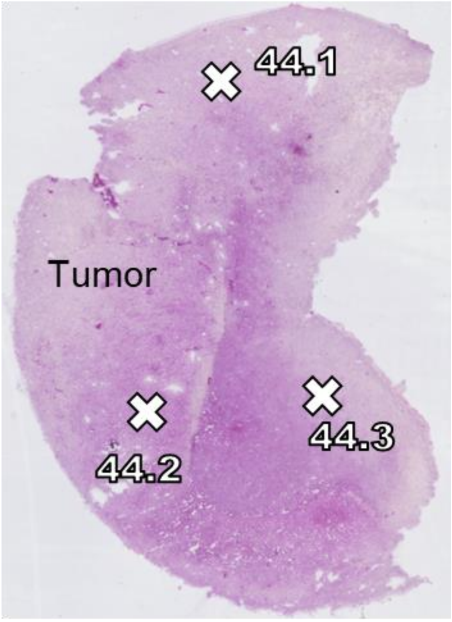

44.1
Tumor
44.2
44.3

39

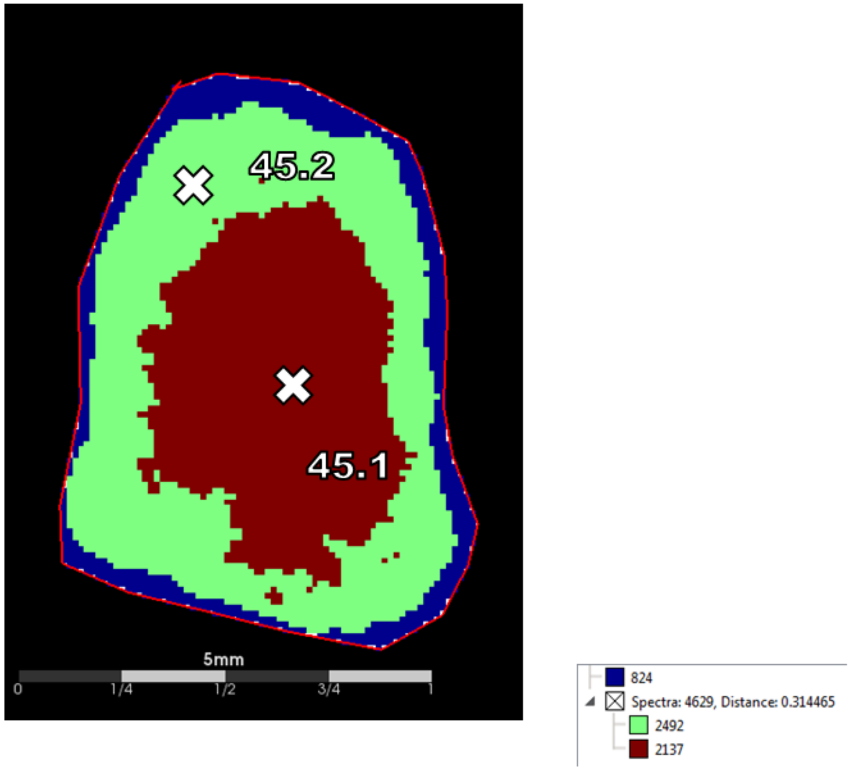
45.2
45.1
5mm
0
1/4
1/2
3/4
1
824
Spectra: 4629, Distance: 0.314465
2492
2137

MVP+
tumor

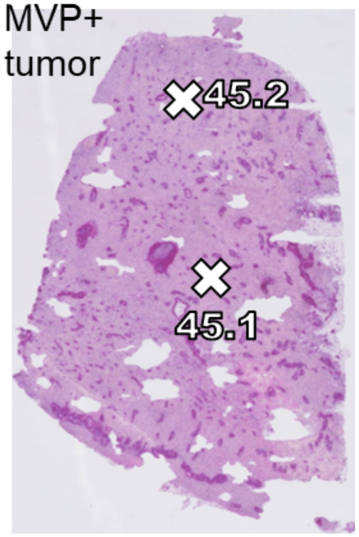
45.2
45.1

40

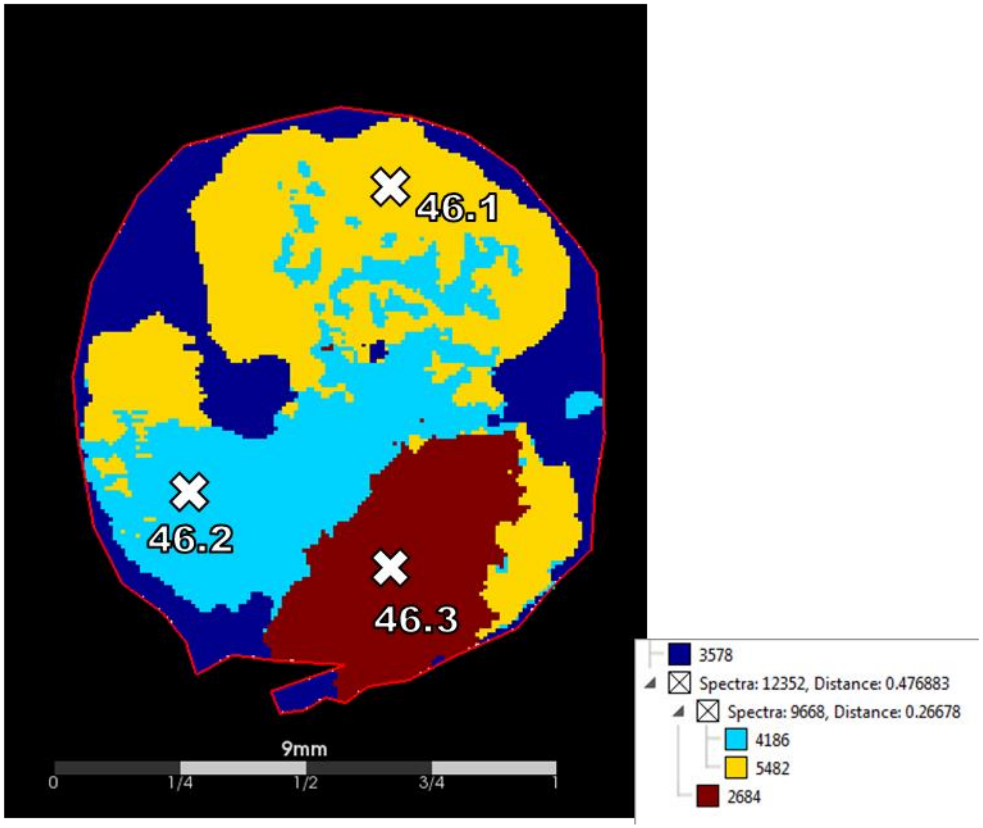
46.1
46.2
46.3
9mm
0
1/4
1/2
3/4
1
3578
Spectra: 12352, Distance: 0.476883
Spectra: 9668, Distance: 0.26678
4186
5482
2684

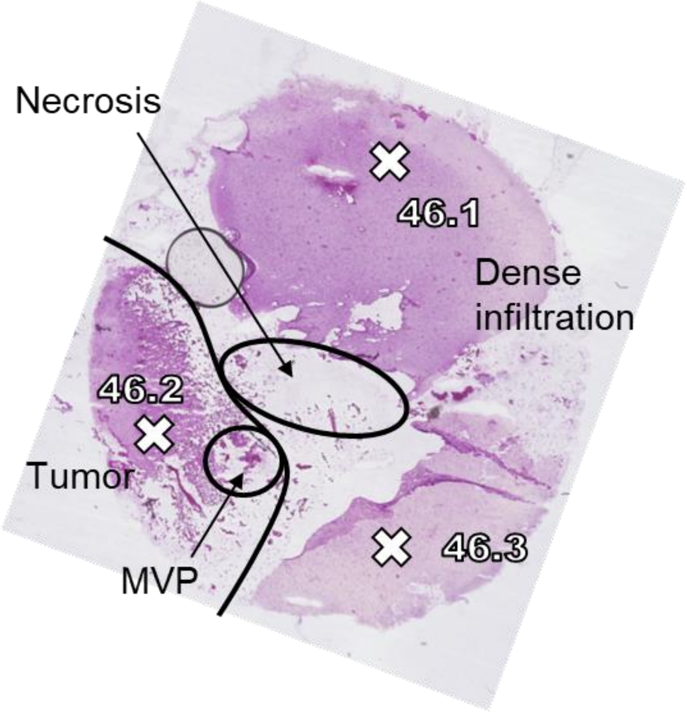
Necrosis
46.1
Dense
infiltration
46.2
Tumor
MVP
46.3

41

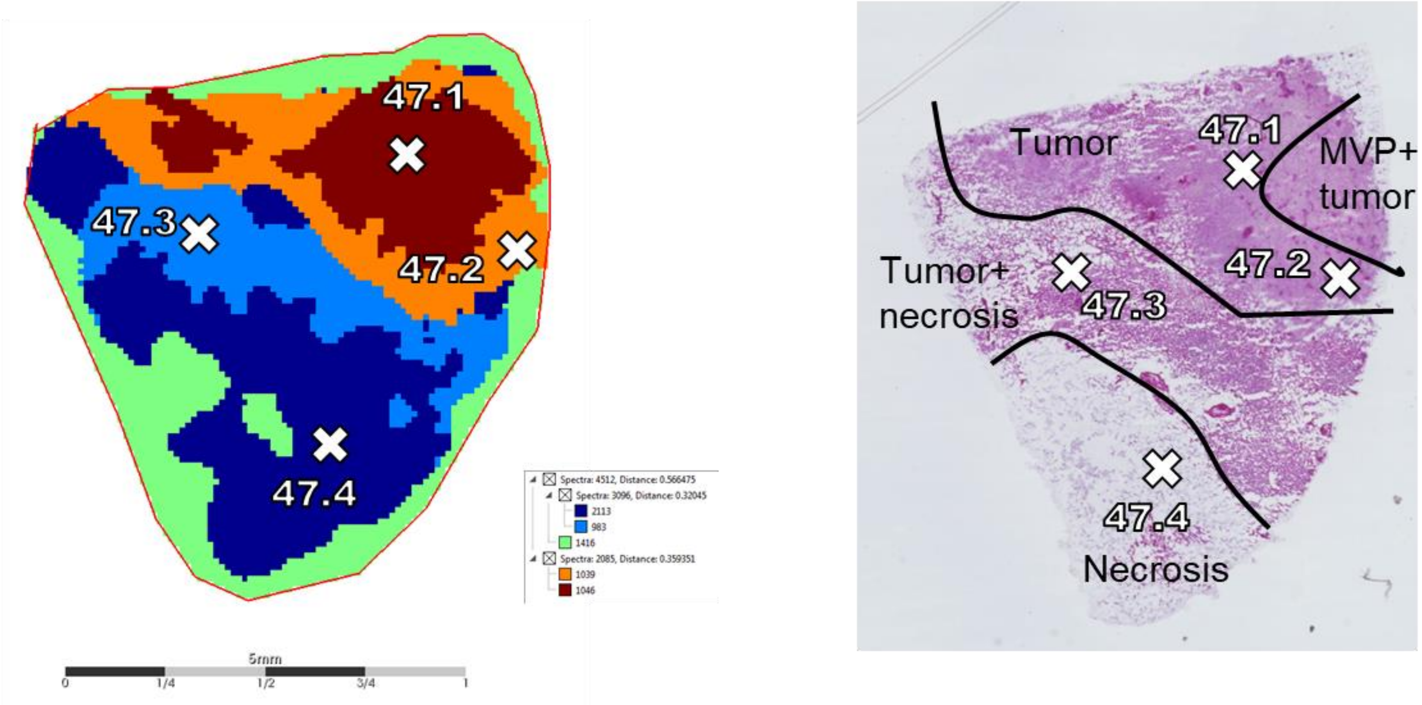

47.1
47.3
47.2
47.4
Spectra: 4512, Distance: 0.566475
Spectra: 3096, Distance: 0.32045
2113
983
1416
Spectra: 2085, Distance: 0.359351
1039
1046
5mm
0
1/4
1/2
3/4
1
Tumor
47.1
MVP+
tumor
Tumor+
necrosis
47.3
47.2
47.4
Necrosis

42

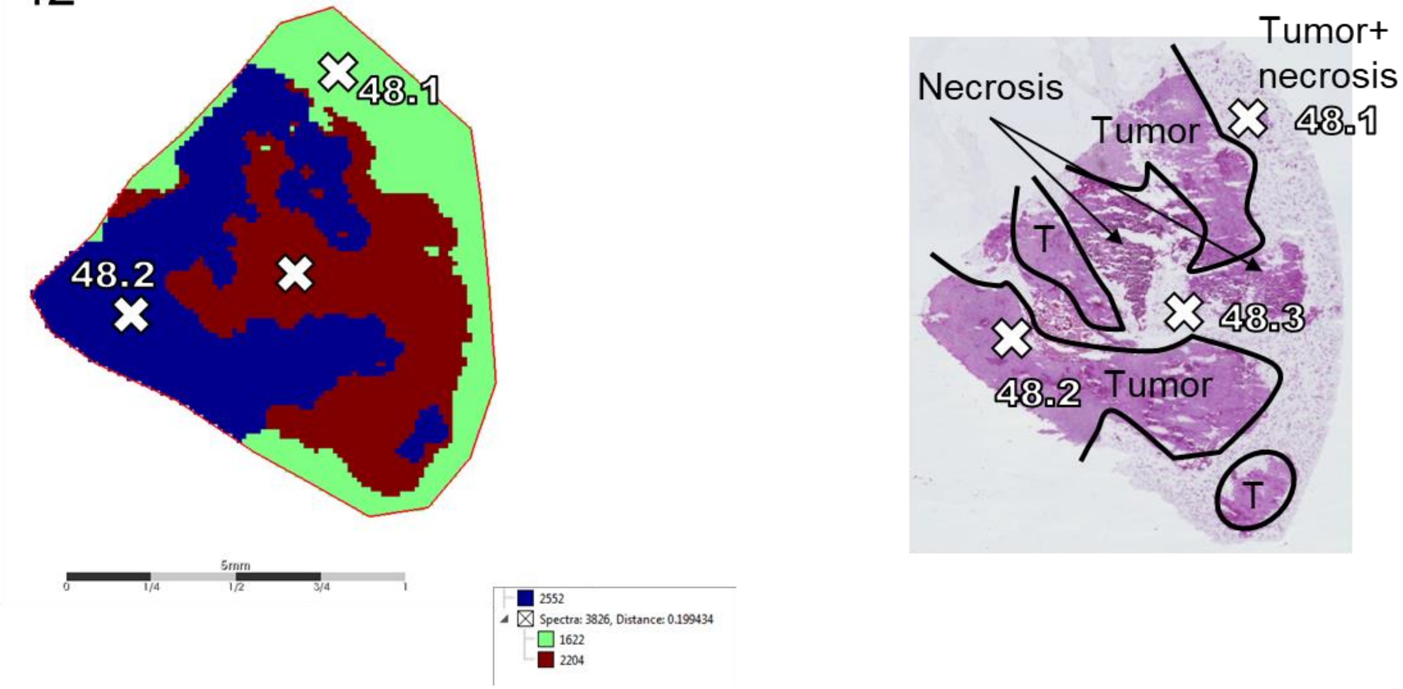
48.1
48.2
5mm
0
1/4
1/2
3/4
1
2552
Spectra: 3826, Distance: 0.199434
1622
2204
Tumor+
necrosis
48.1
Necrosis
Tumor
T
48.3
48.2
Tumor
T

43

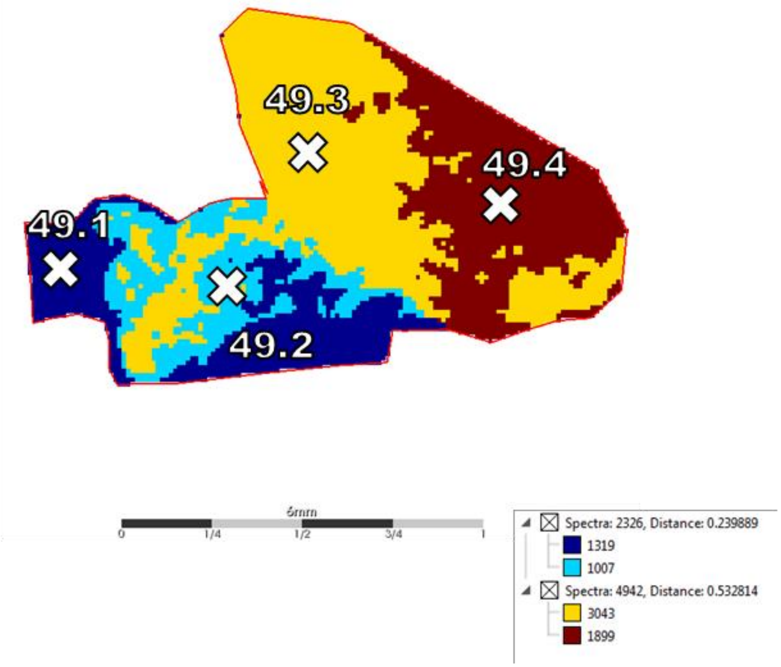
49.3
49.4
49.1
49.2
6mm
0
1/4
1/2
3/4
1
Spectra: 2326, Distance: 0.239889
1319
1007
Spectra: 4942, Distance: 0.532814
3043
1899

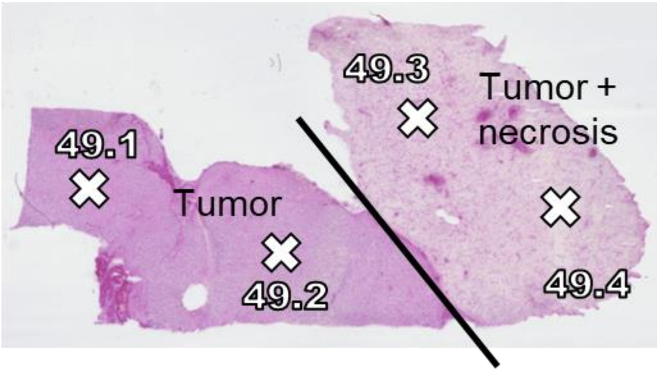
49.3
Tumor +
necrosis
49.1
Tumor
49.2
49.4

44

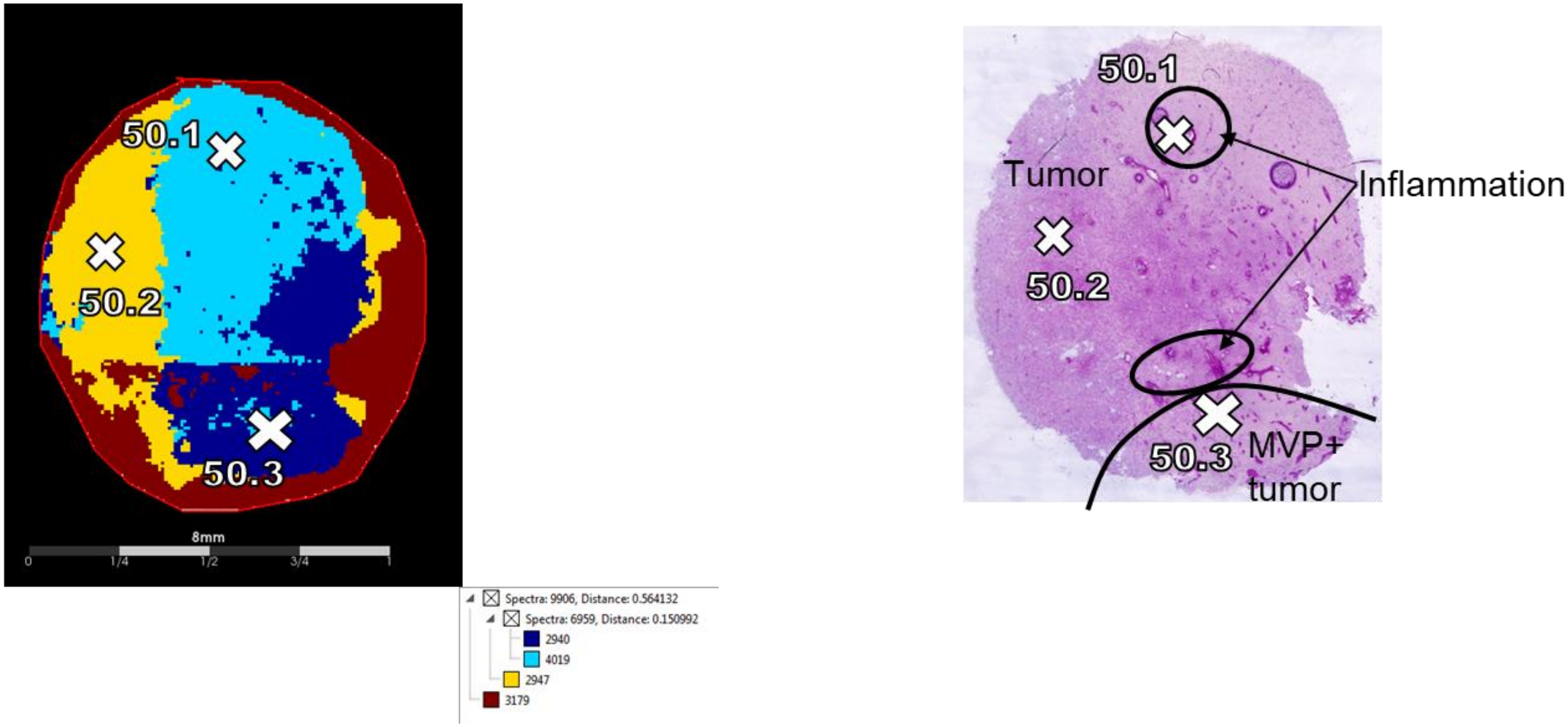
50.1
50.2
50.3
8mm
0
1/4
1/2
3/4
1
Spectra: 9906, Distance: 0.564132
Spectra: 6959, Distance: 0.150992
2940
4019
2947
3179
50.1
Tumor
Inflammation
50.2
50.3
MVP+
tumor

45

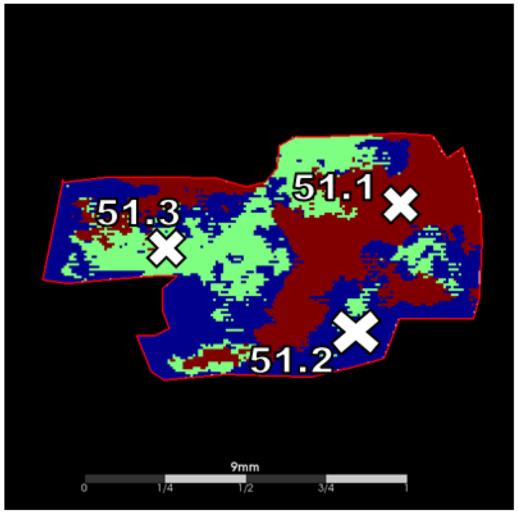
51.1
51.3
51.2
9mm
0
1/4
1/2
3/4
1

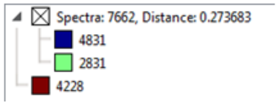
Spectra: 7662, Distance: 0.273683
4831
2831
4228

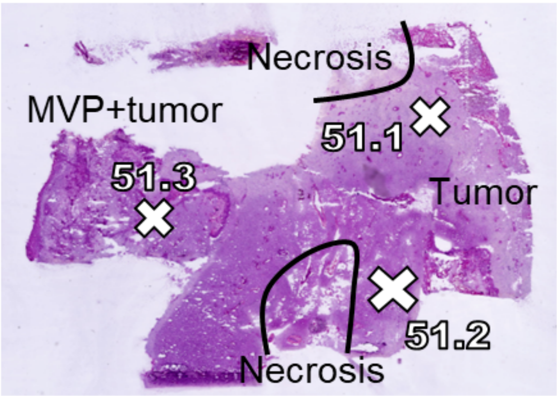
Necrosis
MVP+tumor
51.1
51.3
Tumor
51.2
Necrosis

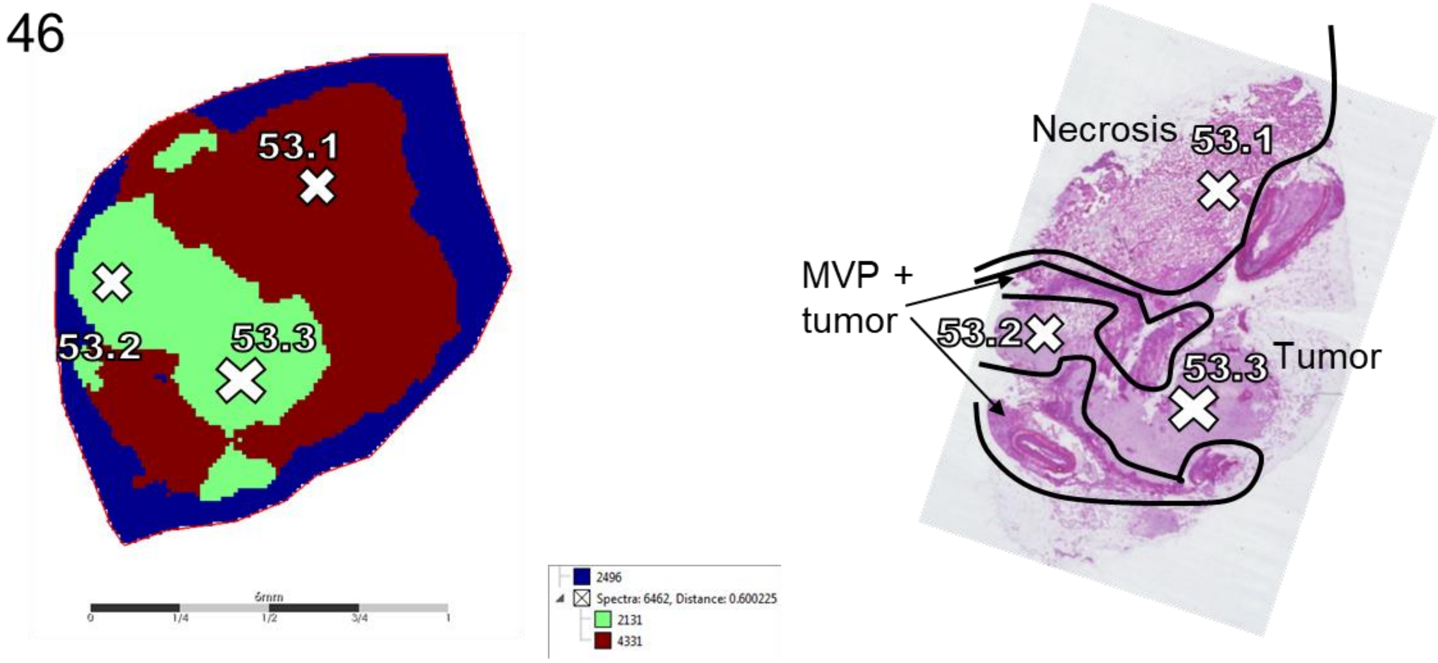

Supplementary Figure 2: Individual segmentation of 46 tumors by MALDI-MSI and comparison with pathologist annotations.

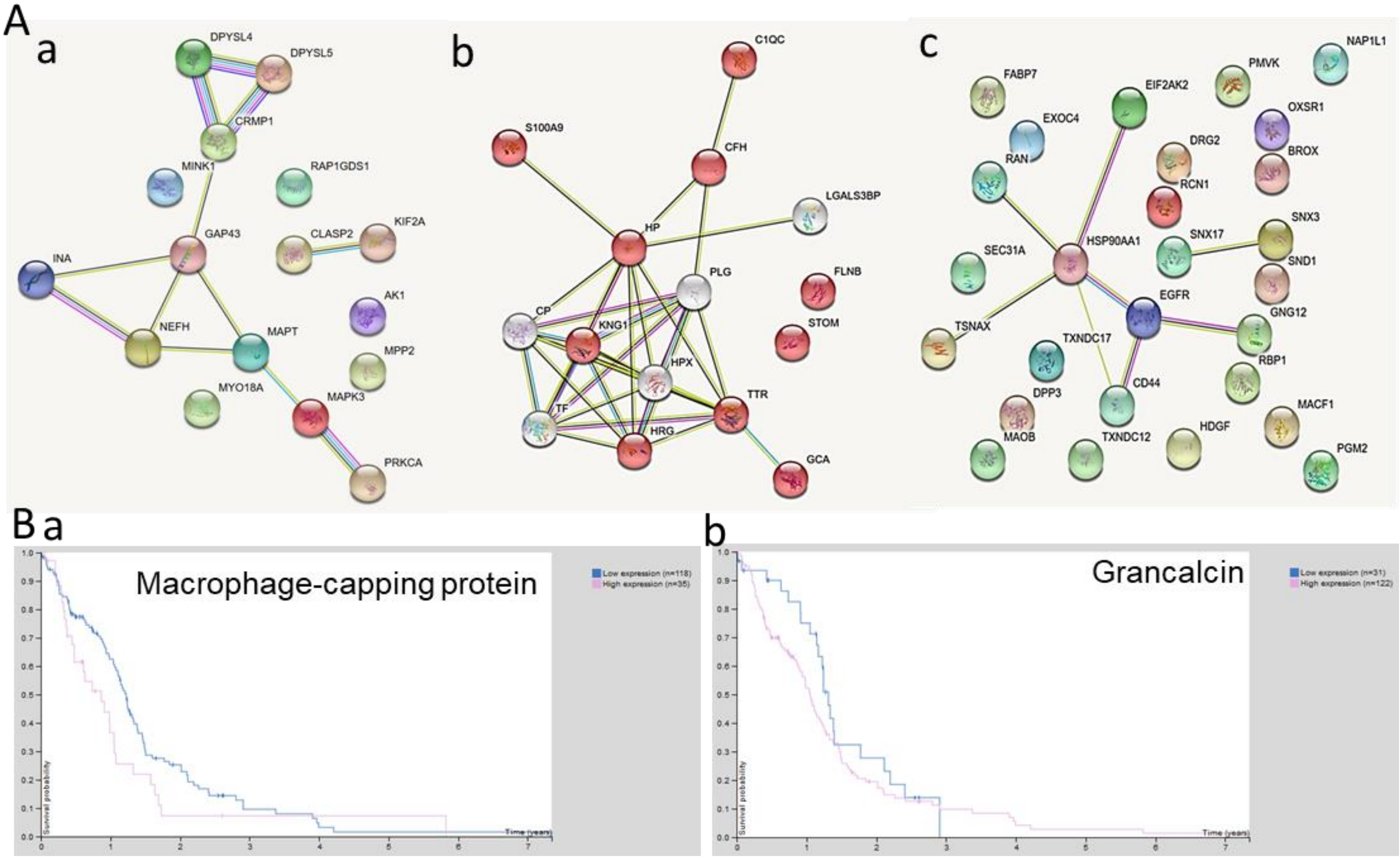

Supplementary Figure 3. String analysis of overexpressed proteins in each of the three proteomic groups and relation to patient survival **A. a)** Analysis of proteins overexpressed in group A shows involvement in axon guidance. **b)** Proteins overexpressed in group B and mainly involved in complements, coagulation cascade and, inflammation **c)** Analysis of overexpressed proteins in group C shows a network of proteins involved in Epstein Barr infection. **B.** Correlation between Grancalcin and CAPG expression and glioma patient survival according to the TGCA data. Patients were divided based on level of expression into “low” or “high” (n = 153 patients).

a

	Pr > Chi-Square
**Karnofsky indice at diagnostic 0-80 vs. 90-100**	0.1051
**MGMT**	0.0882
**Quality of resection complete, subtotal vs. partial, biopsies**	0.7666

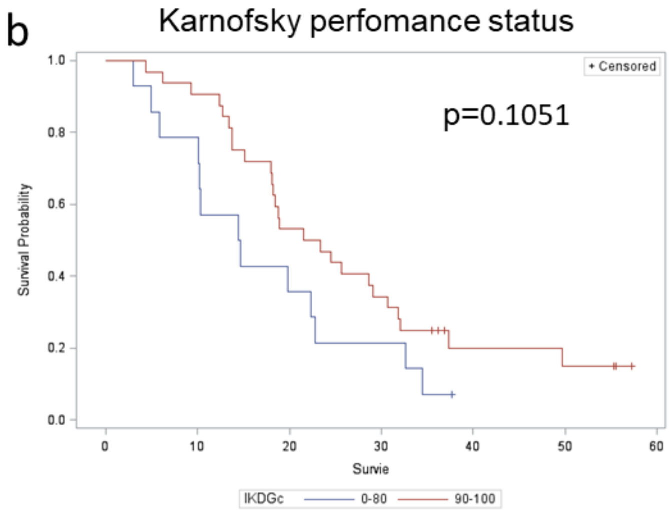

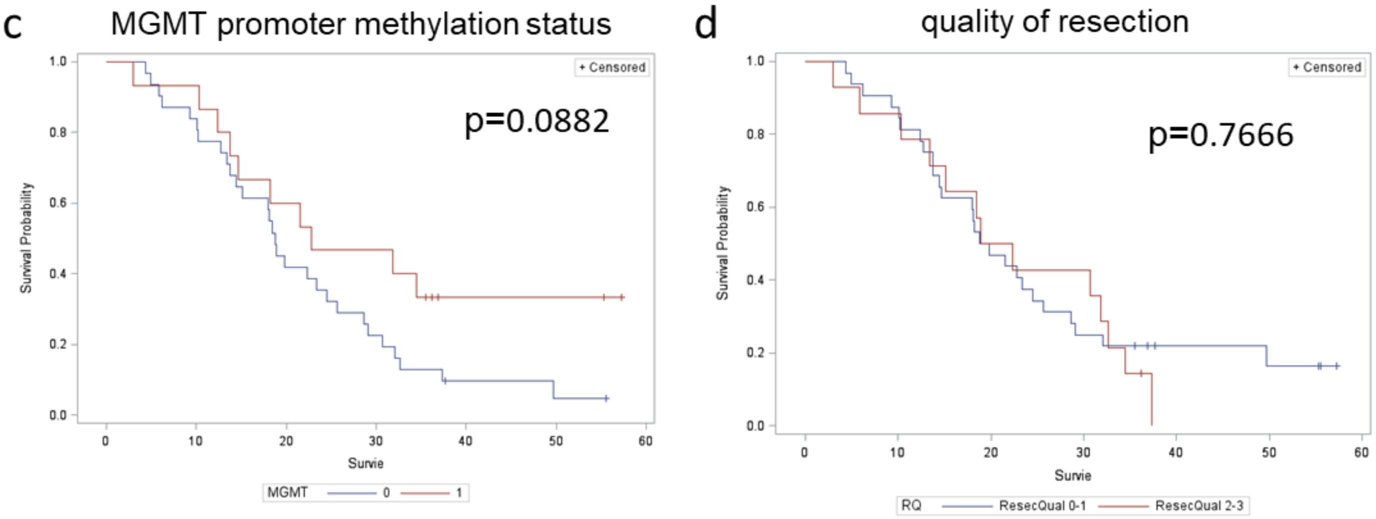

Supplementary Figure 4. Survival curves of all patients according to the Karnofsky performance status, MGMT promoter methylation status, and quality of resection. (a) Clinicopathological factors description (χ2-test). Survival curves (Kaplan-Meier Analysis) of patients according to the Karnofsky performance status (b), MGMT status (c), and resection quality (d). n = 46 patients. The lifetest procedure was used for the statistical analyses (log-rank test, Wilcoxon test).

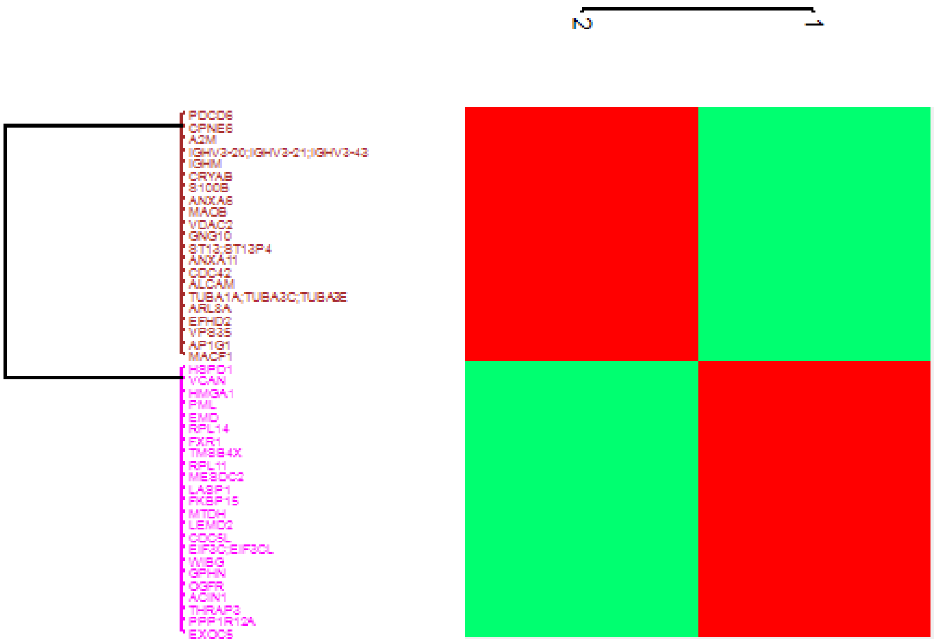

Supplementary Figure 5. Heatmap of the proteins significantly overexpressed in either of the 2 OS clusters (cluster 1 and cluster 2) with a *p* <0.05. Heatmap obtained from the spatially resolved proteomic data which significatively showing overexpression of proteins correlating with the 2 OS clusters (cluster 1 and cluster 2) presenting identified in the patient prospective cohort with a *p*< 0.05. This heatmap only includes the RefProts and does not include the 5 AlProts with a *p* <0.05. Statistical analyses were performed using ANOVA.

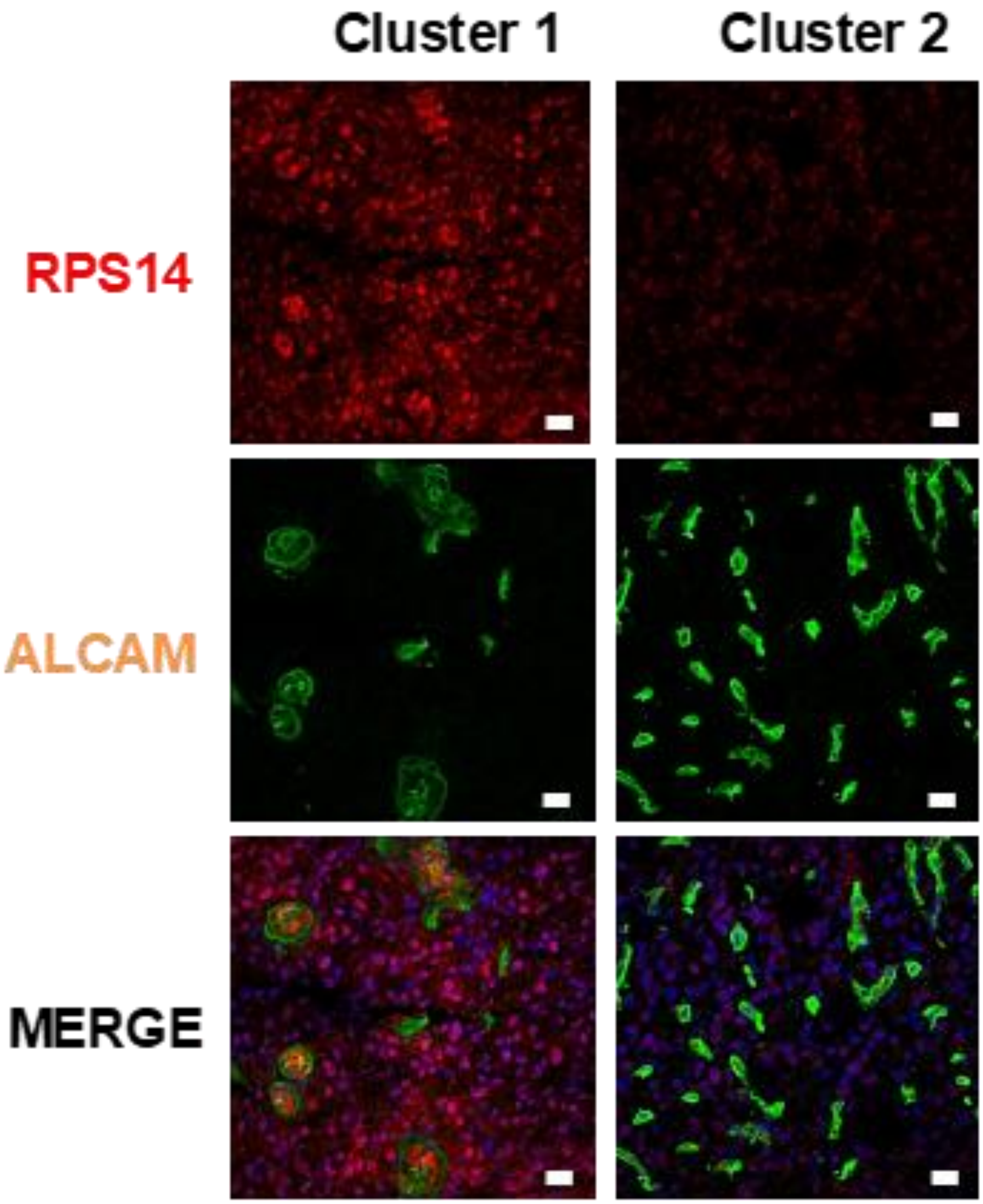

Supplementary Figure 6. Validation immunohistochemistry of RPS14 and ALCAM markers. Representative fluorescence images of RPS14 and ALCAM in the two OS clusters of patients. ALCAM is associated with a bad prognosis while RPS14 is related to a good prognosis. Images were acquired with a confocal microscope at 40x magnification. The experiment was repeated on representative tissues of 23 patients for the prospective cohort and on representative tissues of 50 patients for the validation cohort. For each tissue, 3 to 4 images were taken for quantification. scale bar = 20 µm

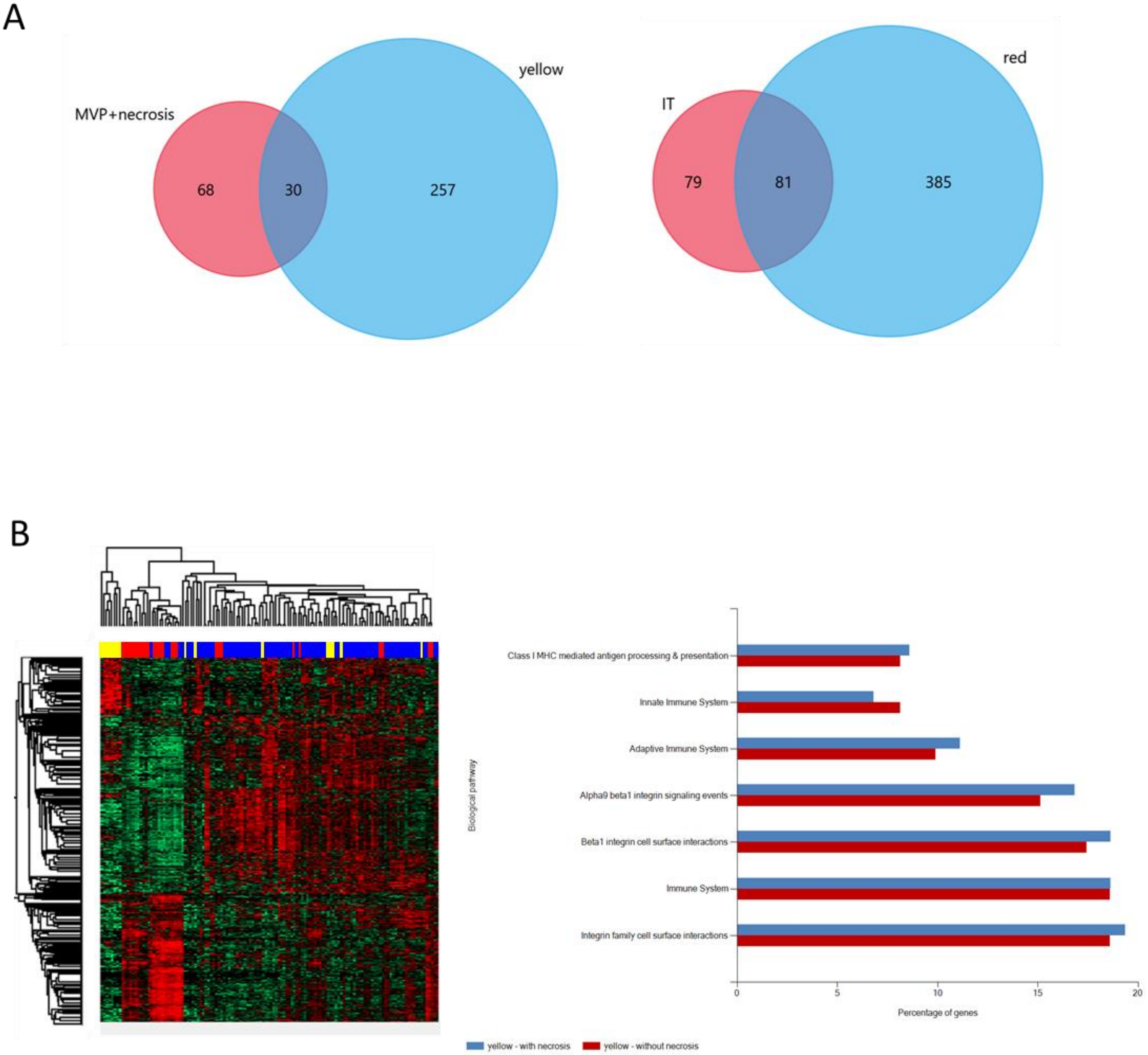

Supplementary Figure 7. Impact of histological features on proteomics analysis. **A. Comparative analysis with the study of Lam et al. (2022)[1].** Venn Diagrams comparing the number of common and differential proteins either in group B (yellow) vs the histological region "MVP+necrosis" of the study of Lam *et al.* or in group A (red) vs the histological region named infiltrated tumor (IT) of the study of Lam *et al*. **B. Proteomics analysis without the micro-extracted points in the necrotic region**. Heatmap of proteins with different regulation profiles as determined by label-free quantification in the three groups without the proteins coming from the necrotic region and comparative analysis of the biological pathways enriched in group B (yellow) with or without the necrotic region.

Supplementary References

[1] Lam, K. H. B. et al. Topographic mapping of the glioblastoma proteome reveals a triple-axis model of intra-tumoral heterogeneity. Nat. Commun. 13, (2022).
