# Supplementary material for: Spatial analysis of the glioblastoma proteome reveals specific molecular signatures and markers of survival
Source: Nat Commun. 2022 Nov 4;13:6665. doi: 10.1038/s41467-022-34208-6 (PMC9636229; doi:10.1038/s41467-022-34208-6)
Supplement: Supplementary file 3 — Description of Additional Supplementary Files [file 41467_2022_34208_MOESM3_ESM.pdf]

Description of Additional Supplementary Files

File Name: Supplementary Data 1
Description: Total matrix extraction from the Perseus file containing proteomics identification

File Name: Supplementary Data 2
Description: List of overexpressed proteins from the clustering of groups A, B, and C

File Name: Supplementary Data 3
Description: Pathways analysis on the different regions

File Name: Supplementary Data 4
Description: A list of 282 of the identified proteins correspond to genes involved in glioma in the TGCA database. These proteins were identified from the entire proteomic dataset of the 147 samples

File Name: Supplementary Data 5
Description: Source data for Integrating proteomics and survival data
