# Supplementary material for: Spatial analysis of the glioblastoma proteome reveals specific molecular signatures and markers of survival
Source: Nat Commun. 2022 Nov 4;13:6665. doi: 10.1038/s41467-022-34208-6 (PMC9636229; doi:10.1038/s41467-022-34208-6)
Supplement: Supplementary file 5 — Reporting Summary [file 41467_2022_34208_MOESM5_ESM.pdf]

nature portfolio

Corresponding author(s): Marie Duhamel, Emilie Le Rhun, Isabelle Fournier and Michel Salzet

Last updated by author(s): Oct 15, 2022

Reporting Summary

Nature Portfolio wishes to improve the reproducibility of the work that we publish. This form provides structure for consistency and transparency in reporting. For further information on Nature Portfolio policies, see our Editorial Policies and the Editorial Policy Checklist.

Statistics

For all statistical analyses, confirm that the following items are present in the figure legend, table legend, main text, or Methods section.

n/a	Confirmed	
☐	☒	The exact sample size (*n*) for each experimental group/condition, given as a discrete number and unit of measurement
☐	☒	A statement on whether measurements were taken from distinct samples or whether the same sample was measured repeatedly
☐	☒	The statistical test(s) used AND whether they are one- or two-sided<br>*Only common tests should be described solely by name; describe more complex techniques in the Methods section.*
☒	☐	A description of all covariates tested
☒	☐	A description of any assumptions or corrections, such as tests of normality and adjustment for multiple comparisons
☐	☒	A full description of the statistical parameters including central tendency (e.g. means) or other basic estimates (e.g. regression coefficient) AND variation (e.g. standard deviation) or associated estimates of uncertainty (e.g. confidence intervals)
☐	☒	For null hypothesis testing, the test statistic (e.g. *F*, *t*, *r*) with confidence intervals, effect sizes, degrees of freedom and *P* value noted<br>*Give P values as exact values whenever suitable.*
☒	☐	For Bayesian analysis, information on the choice of priors and Markov chain Monte Carlo settings
☒	☐	For hierarchical and complex designs, identification of the appropriate level for tests and full reporting of outcomes
☒	☐	Estimates of effect sizes (e.g. Cohen's *d*, Pearson's *r*), indicating how they were calculated

Our web collection on statistics for biologists contains articles on many of the points above.

Software and code

Policy information about availability of computer code

Data collection	For MALDI-MSI, FlexControl v3.3 (Build108) and FlexImaging v2.1 were used. For proteomic analysis, Exactive Series 2.9 and Xcalibur 4.1.
Data analysis	MALDI-MSI analysis was performed using Scils Lab 2019. Protein identification was performed with MaxQuant (Version 1.5.3.30) and statistical analysis with Perseus (version 1.6.0.7) and SAS Software (version 9.4). . For System biology analyses we used PANTHER Classification System (v 14.1), Funrich (v 3.1.3), STRING database (v 11.0), Cytoscape (v 3.7.2) Elsevier's Pathway Studio (v 11.0). SpiderMass Analysis was done using Abstract Model Builder (v 0.9.2092.0). For Aternative protein, Proteome Discoverer (v2.3) was used for identification. For Immunohistochemistry analyses, ImageJ (v1.53) was used.

For manuscripts utilizing custom algorithms or software that are central to the research but not yet described in published literature, software must be made available to editors and reviewers. We strongly encourage code deposition in a community repository (e.g. GitHub). See the Nature Portfolio guidelines for submitting code & software for further information.

Data

Policy information about availability of data

All manuscripts must include a data availability statement. This statement should provide the following information, where applicable:

- Accession codes, unique identifiers, or web links for publicly available datasets
- A description of any restrictions on data availability
- For clinical datasets or third party data, please ensure that the statement adheres to our policy

The data generated in this study including MS raw files, MaxQuant files, and annotated MS/MS datasets have been deposited to the ProteomeXchange Consortium Via PRIDE partner repository with the accession code PXD016165 [http://proteomecentral.proteomexchange.org/cgi/GetDataset?ID=PXD016165]. The public data used in this study were downloaded from The Cancer Genome Atlas Program (TCGA) and are accessible at the Data Coordinating Center (DCC) for public access [http://cancergenome.nih.gov/] and the Human Protein Atlas [https://www.proteinatlas.org/] Database containing Human proteins sequences is accessible at Uniprot [https://www.uniprot.org/] and at OpenProt [https://openprot.org/] for Alternative proteins. The remaining data are available within the Article, Supplementary Information or Source Data file. Source data are provided with this paper.

Human research participants

Policy information about studies involving human research participants and Sex and Gender in Research.

Reporting on sex and gender	In this study, the findings apply to both sexes. Indeed, for the prospective cohort of 46 patients, 15 patients were female while 31 were male. We did not make any differences for the analysis of the data since the clustering we obtained by MALDI-MSI was not based on sex differences.
Population characteristics	Patients were adult, had no medical history of other cancers or previous cancer treatment, no known genetic disease potentially leading to cancer and no neurodegenerative disease. No other covariate characteristics were considered.
Recruitment	Patients with newly diagnosed glioblastoma were prospectively enrolled between September 2014 and November 2018 at Lille University Hospital, France. Tumors samples were processed within 2 hours after sample extraction in the surgery room to limit the risk of degradation of proteins
Ethics oversight	Our research complies with all relevant ethical regulations. Approval of the study protocol was obtained from the research ethics committee (ID-RCB 2014-A00185-42) before the initiation of the study. The study adhered to the principles of the Declaration of Helsinki and the Guidelines for Good Clinical Practice and is registered at NCT02473484. Informed consent was obtained from patients. Participants didn’t receive any compensation.

Note that full information on the approval of the study protocol must also be provided in the manuscript.

Field-specific reporting

Please select the one below that is the best fit for your research. If you are not sure, read the appropriate sections before making your selection.

☒ Life sciences ☐ Behavioural & social sciences ☐ Ecological, evolutionary & environmental sciences

For a reference copy of the document with all sections, see nature.com/documents/nr-reporting-summary-flat.pdf

Life sciences study design

All studies must disclose on these points even when the disclosure is negative.

Sample size	No sample-size calculation was performed. A total of 46 tumor samples were used for mass spectrometry analysis after validation by a pathologist as representative glioblastoma tumors. After MALDI-MSI analysis and based on sample heterogeneity, 147 ROIs were selected (2 to 5 ROIs per tumor sample). A cohort of 50 independant tumors was used used for ortogonal validation by immuno-histochemistry.
Data exclusions	No data were excluded
Replication	MALDI-MSI was performed on 46 tissue samples and grouping was confirmed by SpiderMass analysis, Proteomics analysis was performed in duplicate or triplicate sampling for each regions in each tissue section corresponding to a total of 147 ROI. Hierarchical clustering of proteomic dataset and immunofluorescence analysis confirmed the reproducibility.
Randomization	All sample were anaonymized and randomly analyzed. No experimental groups were allocated. Groups have been deternimed only by the statistical analysis using proteomics data. The tumors were segmented into different molecular regions based on statistical clustering of the MALDI-MSI data and this segmentation was used for proteomic clustering.
Blinding	We did blind experiments for MALDI-MSI without prior knowledge of grouping. Sample were analyzed randomly. We have also performed a first proteomic clustering without a priori. For IHC, we have performed the fluorescent intensity quantification without knowledge of survival of the patients.

Reporting for specific materials, systems and methods

We require information from authors about some types of materials, experimental systems and methods used in many studies. Here, indicate whether each material, system or method listed is relevant to your study. If you are not sure if a list item applies to your research, read the appropriate section before selecting a response.

Materials & experimental systems

n/a	Involved in the study
☐	☒ Antibodies
☒	☐ Eukaryotic cell lines
☒	☐ Palaeontology and archaeology
☒	☐ Animals and other organisms
☐	☒ Clinical data
☒	☐ Dual use research of concern

Methods

n/a	Involved in the study
☒	☐ ChIP-seq
☒	☐ Flow cytometry
☒	☐ MRI-based neuroimaging

Antibodies

Antibodies used

We used the following primary antibodies: ALCAM (R&D Systems; AF1172, polyclonal, lot IQV0119061, 1/40 dilution), RPS14 (Invitrogen, PA5-53823, polyclonal, lot A118773, 1/100 dilution), ANXA11 (OriGene, CF500950, clone OTI1C6, lot F001, 1/100 dilution), PPP1R12A (Invitrogen, PA5-79857, polyclonal, lot V13082589, 1/250 dilution), ANXA6 (Abcam, polyclonal, ab31026, 1/50), MAOB (Abbexa abx431047, polyclonal, 1/100), IGHM (Abcam, ab200541, monoclonal IM260, 1/50), HSPD1 (Abcam, ab53109, polyclonal, 1/200), LASP1 (Santa Cruz Biotechnology, sc374059, monoclonal G-7, 1/50), CFH (Abcam, ab115290, polyclonal, 1/200). We used Alexa Fluor-conjugated secondary antibodies (Life Technologies, 1/200, polyclonal, donkey anti-rabbit 555 A31572, donkey anti-mouse 647 A31571, donkey anti-goat 488 A11055, donkey anti-mouse 488 A21202).

Validation

All antibodies used in the study were validated by the manufacturer for immunofluorescence/IHC applications on human tissues or cells. ALCAM antibody has been referenced in several publications for its use in IHC applications (human doi: 10.1523/JNEUROSCI.5618-10.2011 and mouse doi: 10.1523/JNEUROSCI.0078-08.2008)

Clinical data

Policy information about clinical studies

All manuscripts should comply with the ICMJE guidelines for publication of clinical research and a completed CONSORT checklist must be included with all submissions.

Clinical trial registration

NCT02473484

Study protocol

https://clinicaltrials.gov/ct2/show/NCT02473484

Data collection

Data management and collection are performed by the Neuro-oncology team (Department of Neurosurgery) of the Lille University Hospital between September 2014 and November 2018. A database specific to the study was created. A simple data entry will be performed.The data are checked using the error messages from the validation programs. Obvious errors are corrected. Other errors, omissions or inconsistencies are reported to the investigating physician for resolution.The database is frozen after final quality control and then exported to the SPSS statistical software according to an automated and validated procedure.

Outcomes

Outcomes were predefined to see if spatially resolved proteomics approach is able to investigate the heterogeneity of gliobliostoma.

Primary outcome measures : To evaluate the correlation between the WHO classification 2007 only established standard diagnostic and classification by mass spectrometry gliomas (lipids, peptides, proteins)

Secondary outcome measures:
- To evaluate the a posteriori discordance between the standard WHO 2007 classification and the mass spectrometric classification of gliomas (lipids, peptides, proteins)
- To assess the concordance between the WHO 2007 classification coupled with genetic analysis and MRI interpretation and the mass spectrometric classification of gliomas (lipids, peptides, proteins)
- To assess the a posteriori discrepancy between the standard WHO 2007 classification coupled with genetic analysis and MRI interpretation and the mass spectrometric classification of gliomas (lipids, peptides, proteins)
- To identify by mass spectrometry markers in tumor and blood samples of glioma prognosis

Primary outcome measures : To evaluate the correlation between the WHO classification 2007 only established standard diagnostic and classification by mass spectrometry gliomas (lipids, peptides, proteins)
